# Ovarian absence: a systematic literature review and case series report

**DOI:** 10.1186/s13048-022-01090-1

**Published:** 2023-01-16

**Authors:** H. Alexander Chen, Alyssa A. Grimshaw, Melissa Taylor-Giorlando, Pavithra Vijayakumar, Dan Li, Miranda Margetts, Emanuele Pelosi, Alla Vash-Margita

**Affiliations:** 1 Yale University School of Medicine, New Haven, CT USA; 2 Yale University, Harvey Cushing/John Hay Whitney Medical Library, New Haven, CT USA; 3 Avery Center for Obstetrics and Gynecology, Westport, CT USA; 4 Department of Obstetrics and Gynecology, University of Rochester Medical Center, Rochester, NY USA; 5 Center for American Indian and Rural Health Equity, Montana State University, Bozeman, MT USA; 6 Centre for Clinical Research, The University of Queensland, Brisbane, QLD 4072 Australia; 7 Department of Obstetrics, Gynecology and Reproductive Sciences, Yale New Haven Hospital, New Haven, CT USA; 8 Yale Department of Obstetrics, Gynecology & Reproductive Medicine, Farnam Memorial Building, 310 Cedar Street, Fl 3, Rm 329, New Haven, CT 06510 USA

**Keywords:** Ovarian agenesis, Müllerian anomalies, Renal agenesis, Gonads, Embryology, Infertility

## Abstract

Ovarian absence is an uncommon condition that most frequently presents unilaterally. Several etiologies for the condition have been proposed, including torsion, vascular accident, and embryological defect. A systematic review was conducted to describe the clinical presentation of ovarian absence, as well as its associations with other congenital anomalies**,** through a systematic search of Cochrane Library, ClinicalTrials.gov, Google Scholar, Ovid Embase, Ovid Medline, PubMed, Scopus, and Web of Science. Exclusion criteria included cases with suspicion for Differences of Sex Development, lack of surgically-confirmed ovarian absence, and karyotypes other than 46XX. Our search yielded 12,120 citations, of which 79 studies were included. 10 additional studies were found by citation chasing resulting in a total 113 cases including two unpublished cases presented in this review. Abdominal/pelvic pain (30%) and infertility/subfertility (19%) were the most frequent presentations. Ovarian abnormalities were not noted in 28% of cases with pre-operative ovarian imaging results. Approximately 17% of cases had concomitant uterine abnormalities, while 22% had renal abnormalities. Renal abnormalities were more likely in patients with uterine abnormalities (*p* < 0.005). Torsion or vascular etiology was the most frequently suspected etiology of ovarian absence (52%), followed by indeterminate (27%) and embryologic etiology (21%). Most cases of ovarian absence are likely attributable to torsion or vascular accidents, despite many references to the condition as “agenesis” in the literature. Imaging may fail to correctly diagnose ovarian absence, and diagnostic laparoscopy may be preferable in many cases as genitourinary anatomy and fertility considerations can be assessed during the procedure. Fertility is likely minimally or not affected in women with unilateral ovarian absence.

**Supplementary Information:**

The online version contains supplementary material available at 10.1186/s13048-022-01090-1.

## Introduction

Unilateral ovarian absence (UOA) is a rare finding with prevalence previously reported as 1 in 11,241 [1]. This condition involves absence of a single ovary, often with partial or complete absence of the ipsilateral fallopian tube and/or adnexa, and occasional concomitant abnormalities. Various proposed etiologies for ovarian absence (OA) fall into either embryological, vascular, or torsion-related hypotheses [2]. The embryological view suggests that UOA results from defects in gonadal embryogenesis, leading to congenital absence of one ovary. The vascular hypothesis maintains that ischemia secondary to a vascular accident accounts for UOA and hypoplasia of adjacent adnexal structures. Finally, the torsion hypothesis states that torsion of the ovary leads to adnexal autoamputation. Though torsion or vascular accidents can occur at any age, events in utero or early development may be, respectively, clinical silent or misattributed (e.g. infantile colic). All three mechanisms may contribute to instances of UOA detailed in prior case reports.

In humans, the genitourinary (GU) system derives from the intermediate mesoderm – a component of the germ layer situated between paraxial and lateral plate mesoderm. Gonadal development begins around week four of gestation when the coelomic epithelium of the intermediate mesoderm proliferates forming the genital ridges on either side of the developing embryo. These genital ridges are somatic components of the gonads which will be colonized by primordial germ cells from the posterior endoderm that form the hindgut around week six [3]. At this stage, the gonads are undifferentiated, or bipotential, and will develop into either testes or ovaries depending on genetic cues [4]. Several genes expressed in the bipotential gonads play important roles in the stabilization of the intermediate mesoderm and proliferation of cells within the gonadal primordia. These include the homeobox genes *Emx2* and *Lhx1/9*, the Wilms’ tumor gene *Wt1*, and steroidogenic factor 1 (*Nr5a1* or *Sf1*), whose ablation in the mouse leads to severe disruption of gonadal formation [5–9].

Sex determination occurs around gestation week six-eight and depends on the expression of the Y-linked gene *SRY* and its downstream target *SOX9*, which activate testis development. Ovarian development, once believed to occur by default, requires the action of several genes including *Foxl2*, *Wnt4*, and *Rspo1* [4]. Animal studies have shown that *Foxl2* and *Wnt4* regulate ovarian development by downregulating *Sox9* expression [10]. In addition, *Foxl2* inhibits *Nr5a1* expression by antagonizing *Wt1* [11]. *Rspo1* upregulates *Wnt4* expression and cooperates with *Wnt4* signaling [12]. Following their development, the ovaries descend from the posterior abdomen to the ovarian fossa around the third month of gestation guided by the gubernaculum. Maldescended ovaries, though uncommon, would be found along this line of descent from the paraspinal posterior abdominal wall to the pelvic brim.

The uterus develops separately. Between week seven-nine, the paramesonephric ducts fuse at the cranial end to form the uterus, and caudally to form the upper two thirds of the vagina [13]. The lateral (unfused) portions of the cranial ducts eventually develop into the fallopian tubes. Therefore, concomitant ovarian anomalies are a rare finding in Müllerian agenesis [14]. *WNT4* mutations have been associated with a minority of Müllerian anomalies cases, and ablation of *Wnt4* in mice disrupts Müllerian duct development and sex determination but does not cause gonadal agenesis [10, 15].

The 1-in-11,241 incidence of UOA is based on two cases observed at a single Malaysian institution in the context of 22,483 gynecological and obstetric surgeries [1]. This number is likely an underestimate since UOA is often asymptomatic. This assessment assumes perfect documentation of incidental findings over thousands of surgeries, prior to the widespread use of electronic medical records. The preponderance of case reports over the last few decades likely represents the adoption of laparoscopy within gynecology (thus increased incidental findings), rather than a change in the true incidence of UOA. Though UOA has been the subject of several literature reviews, no systematic review of the topic has been conducted [16–19]. We endeavored to inform the discussion around the etiology of UOA and raise clinical awareness of the condition through a systematic review of ovarian absence, while presenting two cases of UOA from our institution.

## Methods

The Preferred Reporting Items for Systematic Reviews and Meta-analyses (PRISMA) and Synthesis Without Meta-Analysis (SWiM) were used to guide the reporting in this study and the checklists can be found in Supplementary Table 1 [20, 21]. The protocol has been registered in PROSPERO (CRD42020172466) a priori*.*

### Information sources

A systematic search of the literature was conducted by a medical librarian in Cochrane Library, ClinicalTrials.gov, Google Scholar, Ovid Embase, Ovid MEDLINE, PubMed, Scopus, and Web of Science Core Collection databases to identify relevant articles published from the inception of each database to April 2022. The final searches were performed in all the databases on April 6, 2022. The search was peer-reviewed by a second medical librarian using PRESS (Peer Review of Electronic Search Strategies) [22]. Databases were searched using a combination of controlled vocabulary and free text terms for ovarian anomalies. The search was not limited by publication type or year. Details of the full search strategies are listed in Supplementary Table 2. CitationChaser was used to search the reference lists of included studies to find additional relevant studies not retrieved by the database search [23].

### Study selection

Case studies or series were included if the study reported ovarian absence with surgical confirmation. Studies were limited to cases of ovarian absence described in phenotypical females that had 46 XX karyotype or, when karyotype was not stated, absence of syndromic features suggestive of a genetic condition. Studies describing cases with Differences of Sexual Development (DSD) or those not available in English language were excluded.

Four independent reviewers (HAC, PV, MTG, AAG) assessed citations returned by the literature search in pairs, selected relevant abstracts for full text review, and identified full text articles eligible for inclusion in the final review. The lead investigator (AVM) resolved all identified conflicts at each stage of the review process and reviewed the final list of full-text articles to ensure each was relevant to this study’s objectives.

### Data items and data extraction

Three independent reviewers (HAC, PV, AAG) extracted variables from full texts. These included age of patients, presentation symptom(s), surgical procedure, OA laterality, genetic studies, imaging studies, anatomy of the remaining reproductive tract, renal anatomy, and study design. Data items are listed in Table 1.

**Table 1:** Description of ovarian absence cases with demographic, intraoperative, and clinical data for 113 cases of ovarian absence

	**n**	**%**
Ovarian Absence Cases	113	100
**Age (years), mean ± SD**	22.47	± 14.9
Age Groups *		
Infant (0–2 years)	20	18%
Child (2–18 years)	17	15%
Adult (18 + years)	74	65%
Not specified	2	2%
Suspected Etiology *		
Torsion/Vascular accident	58	52%
Embryological	24	21%
Indeterminant/unclear	31	27%
**Surgical Modality**		
Laparoscopy	65	58%
Laparotomy	37	32%
Autopsy	2	2%
Not specified	9	8%
**Imaging Modalities** ^**a**^		
Ultrasound	62	55%
MRI	15	14%
Hysterosalpingogram	14	13%
CT	15	14%
X-Ray	12	11%
Not specified ^**c**^	33	30%
**Genetic Testing**		
Yes ^**b**^	23	20%
No or not specified	90	80%
**Pre-operative Imaging Congruent with Surgical findings**		
Yes	41	50%
No	16	20%
Not Applicable ^**c**^	25	30%
**Clinical Presentation of Ovarian Absence (or procedure leading to diagnosis)**		
Abdominal/Pelvic pain	34	30%
Infertility/Subfertility	21	19%
Evaluation of mass	18	16%
Amenorrhea	13	12%
Sterilization	7	6%
Abnormal uterine bleeding	5	4%
Congenital abnormality	3	3%
Autopsy	3	3%
Malignancy	2	2%
Ectopic pregnancy	3	2%
Cesarean section	1	1%
Foreign body	1	1%
Urinary tract infection	1	1%
Endometrial hyperplasia	1	1%
**Laterality of Absence**		
Left	56	50%
Right	49	43%
Bilateral	8	7%
**Other Structures Affected** ^**a**^		
Absent fallopian tube	56	50%
Partial fallopian tube	39	35%
Absent round ligament	14	12%
Absent broad ligament	9	8%
Absent infundibulopelvic ligament	5	5%
Absent utero-ovarian ligament	1	1%
None	4	4%
Not specified /unclear	10	9%
**Other Structures Affected (laterality, to absent ovary)**		
Ipsilateral	91	81%
Bilateral	6	5%
Not Stated	16	14%
**Uterine Anatomy **^**a**^		
Normal uterus	66	58%
Hypoplastic/Rudimentary uterus	8	7%
Uterine Leiomyoma	3	3%
Abnormal uterine variations	19	17%
Unicornuate	11	58%
Absent (non-surgically)	4	21%
Mature cystic teratoma on anterior uterine wall	1	5%
Septate	1	5%
Arcuate	1	5%
Sagittal sulcus on fundus	1	5%
Not specified	19	17%
**Endometriosis**		
Present	7	6%
Absent or not specified	106	94%
**Kidney anatomy**		
Normal	58	51%
Different variations ^**a**^	25	22%
Renal agenesis	18	72%
Ectopic kidney	3	12%
Nephrolithiasis	2	8%
Cystic kidney	1	4%
Pyelitis	1	4%
Not specified	30	27%
**Renal Abnormality Laterality**		
Ipsilateral (to the ovary)	21	84%
Contralateral	3	12%
Bilateral	1	4%
**Mass/pathology report**		
**Mass present**	28	25%
Ovarian tissue	10	36%
Non-ovarian tissue	10	36%
Indeterminant (possible ovarian)	7	25%
Mass not removed	1	4%
**Mass absent**	85	75%

^*^ (*p* < 0.001)

^a^ sum% > 100%, as some patients had multiple studies and clinical findings

^b^ 46,XX karyotype was reported in all cases that mentioned genetic testing and met the inclusion criteria. Cases with chromosomal abnormalities (e.g. 45,XO) were excluded

^c^ encompasses manuscripts that did not mention imaging findings (not stated), or reported non-tuboovarian imaging studies (e.g. chest X-ray)

### Assessment of risk of bias

Manuscripts were assessed for quality of evidence based on the Oxford Centre for Evidence-Based Medicine (OCEBM): Levels of Evidence. This scale grades manuscripts from 1 (highest) to 5 (lowest) according to the level of clinical evidence that can be derived from their study design. Randomized control trials are designated 1, cases-series are 4, while case reports and expert opinions are graded 5. Case reports were subsequently assessed using the JBI Critical Appraisal Checklist for Case Reports [24].

### Data synthesis

Statistical analysis was performed using SAS software (v9.4, SAS Institute. Cary, NC, USA) and Microsoft Excel (Microsoft Office, v16.16.27). We performed quantitative, qualitative, and formal narrative syntheses of data extracted from the case reports and literature reviews included in our systematic review of OA. We performed univariate analysis on the categorical variables and reported their frequencies and relative percentages. We conducted chi-square tests to evaluate the relationships between several categorical variables. During the data analysis process, we used the fixed-effect assumption. Publications included in this systematic review were primarily case reports and case series, and there was significant variability in reporting of case details.

## Cases

### Case #1

**Fig. 1:**
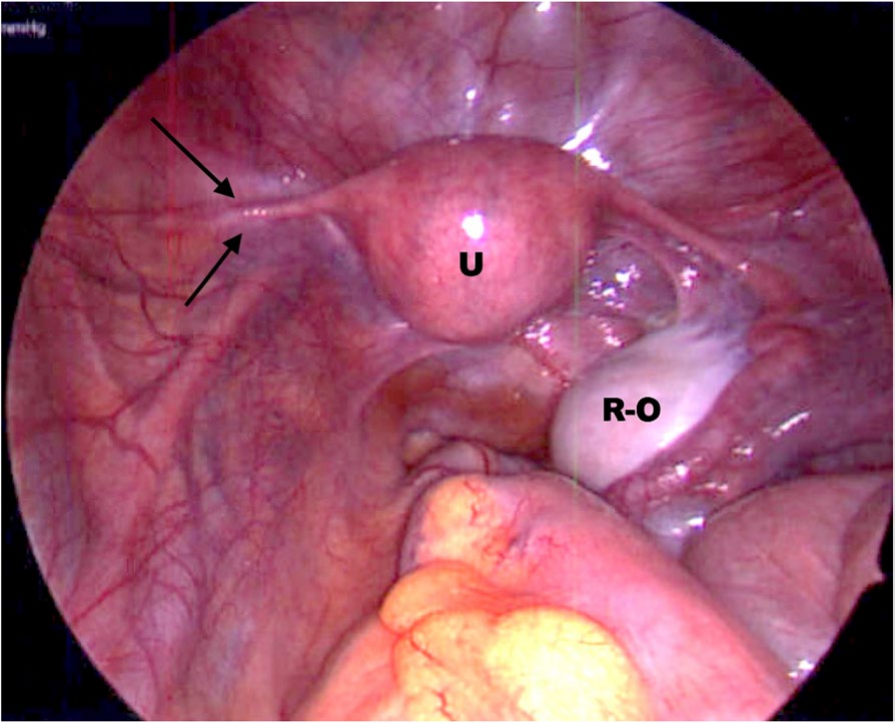
Intraoperative laparoscopic view of pelvis from case 1. U = uterus, R-O = right ovary, arrows point to a partial left fallopian tube with absent left ovary

A 15-year-old postmenarchal girl presented to Yale New Haven Children’s Hospital for a diagnostic laparoscopy due to chronic abdominal and pelvic pain. Menarche occurred at age 12. She reported regular menses with normal flow and acyclic pelvic and flank pain. She denied being sexually active. Past medical and surgical history were significant for ADHD, dyslexia, nephrolithiasis, constipation, tonsillectomy and adenoidectomy. There was no documented family history of endometriosis, but there was a suspicion for endometriosis necessitating oophorectomy in the maternal grandmother. Physical exam was notable for tenderness with deep palpation in the left upper and lower abdominal quadrants. She had age-appropriate secondary sexual characteristics with Tanner stage 5 breasts and Tanner stage 4 pubic hair. The external inspection of the genitalia revealed normal labia, clitoris, prepuce, external urethra meatus, and annular hymen. Renal ultrasound demonstrated left-sided nonobstructing kidney stones. The transabdominal pelvic ultrasound was unremarkable. Three prior ultrasounds reported normal-appearing ovaries bilaterally. The patient was started on continuous combined ethinyl estradiol-progesterone oral formulation to suppress menses due to suspicion of endometriosis. After four months, she continued to experience pelvic pain and irregular vaginal bleeding. Following a shared decision-making process, the patient and guardian elected to proceed with diagnostic laparoscopy. Surgery revealed a normal appearing uterus with bilateral round ligaments and a normal appearing right fallopian tube leading to an enlarged right ovary. Lesions suspicious for endometriosis were observed and biopsied in the pouch of Douglas, on the sigmoid colon, and the right uterosacral ligament. The left ovary, distal fallopian tube, and utero-ovarian ligament were absent. The left cornua of the uterus had a 3 cm tubular structure which was presumed to be the left fallopian tube remnant. (Fig. 1). The left ureter was clearly observed passing over the external iliac vessels, but no other adnexal structures, including the infundibulopelvic (IP) ligament, were observed. Multiple peritoneal biopsies revealed endometriosis and stage 3 was assigned per American Society for Reproductive Medicine classification [25]. A second gynecology attending physician was consulted intraoperatively and concurred with the incidental finding of the absent left ovary. The patient had an uncomplicated postoperative course. Endometriosis was managed by a 30 mcg-1.5 mg ethinyl estradiol-norethindrone pill in continuous regimen, resulting in amenorrhea and the resolution of both pelvic and flank pain. Postoperative MRI of the abdomen and pelvis did not identify ortho/heterotopic left ovary.

### Case #2

The second patient is a 30-year-old G3P1011 (obstetric history significant for one term delivery of a living child and one ectopic pregnancy) woman with a history of asthma and genital chlamydia infection. Her surgical history was significant for a supraumbilical laparotomy in infancy to retrieve a severed umbilical catheter remnant. She had no other abdominal surgery. She had a history of a right tubal ectopic pregnancy which was successfully treated with methotrexate. The patient presented to the hospital with severe abdominal pain and a positive home pregnancy test. On abdominal exam she had mild suprapubic tenderness with rebound. Her pelvic exam revealed normal appearing labia, clitoris, and external urethral meatus. Speculum exam demonstrated multiparous cervix, without discharge or blood. Bimanual examination revealed a small, anteverted uterus with no masses appreciated in the adnexa bilaterally, and mild tenderness in the posterior fornix. The serum beta-HCG level was 5,519 mIU/mL. The patient had two pelvic ultrasounds. The first revealed normal bilateral ovaries with normal blood flow and a complex cystic right adnexal structure possibly indicative of an ectopic pregnancy. The second ultrasound suggested torsion of the left fallopian tube, a cystic structure with a possible yolk sac in the right adnexa, and no distinct right ovary. Neither ultrasound indicated an intrauterine pregnancy. The patient elected to have diagnostic laparoscopy due to a suspected ectopic pregnancy and adnexal torsion. Operative findings were significant for the minimal adhesive disease and a right tubal ectopic pregnancy. The left ovary and adnexa appeared normal as did the bilateral round ligaments. An ectopic pregnancy was found extruding from the remnant of the right fallopian tube that had dived into the posterior leaf of the broad ligament. Notably, the right ovary, distal 2/3 of the right fallopian tube and the right IP ligament were missing. To verify the absence of the right adnexal structures, dissection of the right pelvic side wall was performed. This revealed normal appearing ureter and iliac vessels, but the right ovary and distal 2/3 of the fallopian tube were not visualized. Of note, a second gynecology attending physician was consulted intraoperatively to confirm the absence of the right ovary. The pathology of the extruded gestational tissue revealed decidualized stromal tissue with implantation site trophoblasts, consistent with an ectopic pregnancy. Postoperative recovery was uneventful.

## Results

### Study selection

**Fig. 2:**
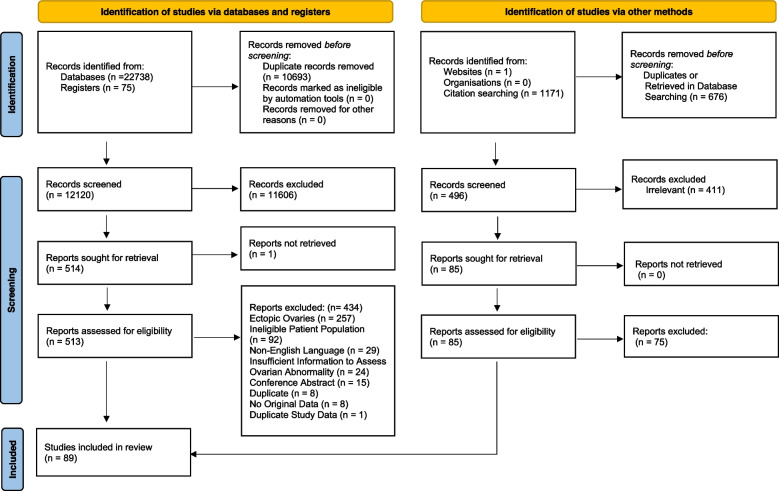
PRISMA flowchart. Additional information on screening methodology and excluded studies can be found in the supplementary material. Adapted from: Page MJ, McKenzie JE, Bossuyt PM, Boutron I, Hoffmann TC, Mulrow CD, et al. The PRISMA 2020 statement: an updated guideline for reporting systematic reviews. BMJ 2021;372: n71. https://doi.org/10.1136/bmj. n71. For more information, visit: http://www.prisma-statement.org/

Database searches resulted in 22,813 citations (Fig. 2). After removing duplicates, 12,120 citations underwent title and abstract screening. Of these, 513 citations met the criteria for full text review. Subsequently, 79 studies met the inclusion criteria for the study. An additional 10 studies were found through reference chasing and searching Google for a total of 89 included manuscripts (Supplementary Table 3). We excluded 434 studies as those presented data on ectopic ovaries, ineligible patient populations, non-English languages, had insufficient information to assess ovarian abnormality, were conference abstracts, were duplicates, had no original data, or presented duplicate study data (Supplementary Table 4).

### Study characteristics

The analysis comprised 89 studies with 113 cases of bilateral and unilateral ovarian absence, all of which were from English-language articles published from 1909 to 2022. All articles were case reports or case series, some of which were accompanied by literature reviews.

### Risk of bias of included studies

10 studies were OCEBM Level 4 (11%) and 79 studies were Level 5 (89%). Using the JBI Critical Appraisal Tool for Case Reports, the overall quality of 89 included records was generally good, with > 90% records scoring “yes” in 6 or more domains. The demographics domain was poorly reported across 59 papers, in most cases this was due to the lack of reporting on race and ethnicity. The detailed quality assessment for each study can be found in Supplementary Table 5 and Supplementary Fig. 1.

### Synthesis of results

The average age of OA diagnosis was 22.47 years (Table 1). A torsion or vascular etiology was the suspected etiology of OA in 58 cases (52%), while an embryologic etiology was suspected in 24 cases (21%). Etiology was undetermined in 31 cases (27%). Ultrasound was the most common imaging technique performed (55%), and laparoscopy was the most common surgical modality (58%). Of the cases that included pre-operative ovarian imaging results, 16/57 (28%) did not mention ovarian abnormalities. Abdominal/pelvic pain (30%) and infertility/subfertility (19%) were the most common presenting symptoms, followed by evaluation of a mass (16%) and amenorrhea (12%). Ovarian absence was most commonly unilateral, with both sides approximately equally affected. Some degree of ipsilateral fallopian tube absence was present in 85% of cases. Approximately 17% of cases had concomitant uterine anomalies, while 22% had renal abnormalities. An additional 10% of cases were comprised of uterine leiomyoma (*n* = 3) or hypoplastic/rudimentary uterus (*n* = 8). The most common uterine anomaly recorded was unicornuate uterus, followed by non-surgical uterine absence. Of note, patients who presented with uterine anomalies were more likely to have a renal abnormality. (*p* < 0.005), and patients with absent fallopian tube were more likely to have renal agenesis (*p* < 0.005).

#### Primary Amenorrhea

A small minority of cases reported bilateral ovarian absence, typically presenting as primary amenorrhea [26, 27]. Mutchinik et al. described an 18-year-old female with primary amenorrhea, hypergonadotropic hypogonadism, bilateral gonadal absence, rudimentary uterus and right fallopian tube with a normal vagina and kidneys [28]. Centromeric Y-chromosome DNA*, SRY* and *ZFY* genes were negative, ruling out the presence of genomic sequences associated with streak gonads or rare intersex conditions [28]. These cases represent a rare congenital anomaly of bilateral OA leading to early diagnosis and profound phenotypical characteristics such as absent pubertal development, delayed bone maturation, primary ovarian insufficiency, and the need for lifelong hormonal supplementation. One patient was noted to have a similar phenotype despite one intact ovary [29].

#### Müllerian anomalies

Approximately 17% of cases of OA had concomitant uterine anomalies.

Two instances of MRKH with 46,XX karyotype and bilateral ovarian absence were observed [30, 31].

#### Concomitant Müllerian and renal anomalies

Osmanagaoglu described a 17-year-old female who presented with amenorrhea with unicornuate uterus, right tuboovarian absence, and ipsilateral renal agenesis [32]. An instance of left UOA with ipsilateral renal agenesis and contralateral hydronephrosis was observed in a patient with MRKH type II [33].

#### Infertility

Infertility/subfertility was the second most common presenting symptom in the diagnosis of the OA, comprising 19% of all cases**.** Kriplani et al. reported a 36-year-old G0 female with secondary amenorrhea and a 14-year history of infertility [34]. Laparotomy revealed a normal uterus, bilateral partial tubes and an omental mass with pathology consistent with ovarian tissue and a dermoid cyst. Sivanesaratnam described a 23-year-old woman whose initial hysterosalpingography (HSG) showed a blocked right tube at the cornual end. Subsequent laparoscopy demonstrated absent right tube and ovary, with no other GU abnormalities on postoperative workup [1]. Peer et al. reported a 27-year-old nulligravid woman with regular menses and history of infertility [35]. Laparotomy revealed an ectopic unilateral ovary within the omentum, as well as contralateral OA. Other cases leading to the diagnosis of the UOA during infertility workup have been described [17, 36].

#### Pelvic masses

Sunku et al. described a 16-year-old female with secondary amenorrhea, synchronous cervical rhabdomyosarcoma, and Sertoli-Leydig cell tumor of the ovary with unilateral tuboovarian absence [37]. A number of cases noted pelvic masses diagnosed concomitantly with UOA with pathologies such as a mucinous cystadenocarcinoma, dermoid cysts, a serous cystadenoma, and fibrous calcified masses consistent with autoamputated ovary [34, 38–46].

#### Endometriosis

Including our case of the adolescent patient, we noted seven reports of UOA with concomitant endometriosis [16, 18, 39, 47–50]. There was one instance of septate uterus, and one separate instance of the ipsilateral renal agenesis [48, 49]. Our adolescent patient had stage 3 endometriosis with UOA and ipsilateral nephrolithiasis. The prevalence of endometriosis in our review (7/110) is comparable to the prevalence among reproductive-age women (~ 10–15%), suggesting a similar prevalence of endometriosis in patients with UOA [51].

## Discussion

Various proposed etiologies for OA fall into either embryological, vascular, or torsion hypotheses [2]. All three mechanisms may explain the findings detailed in prior case reports, and the hypotheses are noncompeting in that each may be true in different instances. However, with distinct etiologies of OA one would also expect distinct natural histories of the condition. We divided cases by plausible etiology based on the case presentation, as well as the original authors’ suggested etiology. Torsion and vascular etiologies were combined as they both result in tissue damage due to the lack of perfusion, and separating the two, post hoc*,* is speculative at best. Cases of unknown etiology largely represent insufficient evidence to rule-in embryological causes rather than to rule-out mechanical/vascular causes. As such, it is likely that many unknown etiology cases are attributable to torsion, and the prevalence of torsion etiology in our review (52%) is understated. Categorizing all unknown cases as torsion gives an upper range of 79%, suggesting torsional etiologies were observed with approximately 2.5–4 times the frequency of embryological etiologies.

While many prior cases label their findings as “agenesis”, the term “absence” is better suited to describing findings that result from mechanical or vascular incidents rather than the failure of the ovary to develop during embryogenesis due to absent primordial tissue (i.e., agenesis). As most cases of UOA do not present with concomitant GU abnormalities, but rather can be explained by infarction and torsion, this suggests *against* agenesis as an etiology for most UOA cases.

### Torsion/vascular

Ovarian torsion leading to autoamputation may be preceded by the formation of an ovarian cyst or other outgrowth that raises the risk of torsion. Torsion, in turn, would typically manifest with nausea, vomiting, and acute-onset abdominal pain. Evidence of scarring and adhesions would also be expected during surgery. While some UOA patients report a history of severe abdominal pain for which they did not seek treatment, others recall no such history [1, 18, 52–54]. In a prior UOA article, Sirisena emphasized the idea that “asymptomatic torsion” is paradoxical – as it necessarily presents as acute abdominal pain [46]. The lack of a painful event in the patient’s history may be explained by torsion while in utero or during infancy when distress is difficult to attribute. Such torsion may lead to autoamputation of the ovary which would then undergo necrosis and calcification, resorption, or possible reimplantation. With reimplantation, one may expect to find histologically verified ovarian remnants in regions outside the normal path of descent. In this sense, the presentation of UOA torsion cases fall along a spectrum, from the initial autoamputation event to the complete degradation or resorption of the ovary, or persistence of a wandering abdominopelvic mass. Instances of rudimentary or hypoplastic ovaries may be a sign of partial autoamputation, falling within this spectrum but outside the scope of this review [2].

Multiple cases in our review support the existence of such a spectrum, consistent with the natural history we proposed above. At one institution, the rate of autoamputation following antenatal ovarian torsion was > 60% [55]. We identified cases of fetuses with ovarian cysts diagnosed in utero with subsequent autoamputation seen in postnatal surgery [56–59]. These cases were found to have calcified or necrotic ovoid masses in the pelvis, most of which had indeterminant tissue origin on histology. We also noted that similar calcified pelvic masses were found incidentally in adult patients, suggesting that these masses may persist for decades following torsion [43, 60]. Several cases of mature cystic teratomas were observed in older adult patients, all of which were suspicious for ovarian torsion [34, 61]. It was undetermined whether these cases resulted from autoamputation of a torsed dermoid, or transformation of previously autoamputated and reimplanted ovarian tissue. Torsion of the remaining ovary in UOA patients, necessitating salpingoophorectomy, may induce primary ovarian insufficiency. Most reported instances of ovarian torsion occur on the right side – possibly because the ovarian ligament is typically longer, and the left side is relatively protected by the sigmoid colon [62]. However, we found no significant difference in UOA laterality overall or in our torsion etiology group.

Overlap between embryological and vascular causes may exist. It has been suggested that vascular compromise to the primitive ovary or the Müllerian duct could occur during elongation and spinalization of the tube during fourth to fifth month of embryologic development, resulting in ovarian aplasia or hypoplasia [63]. The presence of a calcified remnant in the pelvic cavity may suggest torsion-related autoamputation, though this remains speculative [38]. Two cases of OA presented here occurred likely due to an undiagnosed torsion. Both had normal appearing ipsilateral ureters, suggesting against urogenital abnormalities that would imply an embryological cause. The patient in case 1 also received additional imaging that revealed bilateral kidneys. This finding is consistent with a prior review of ovarian autoamputation by Focseneanu et al. that reported a paucity of renal abnormalities across 94 cases [64]. The authors also noted that most reports of UOA featured partial/absent fallopian tubes, and that ovaries and tubes have distinct embryological origins. Because renal findings would be expected if tubal absence was congenital rather than torsional, and their series demonstrated no renal findings [64]. Focseneanu et al. concluded that congenital absence is not a plausible etiology of OA [64]. We would argue that congenital tubal and ovarian absence could be explained by embryological defect that produces concomitant uterine anomalies and ipsilateral renal anomalies as described below.

### Embryological

Cases in our study with suspected embryological origins were all notable for comorbid GU abnormalities– most commonly complete ipsilateral renal agenesis. Uterine abnormalities such as Müllerian agenesis, unicornuate uterus, and hypoplastic/rudimentary uterus were also frequently observed in this group. Bousfiha et al. reports on a 19-year-old female with bilateral ovarian dysgenesis, concomitant uterine aplasia (MRKH), and normal karyotype [31]. Similarly, Kumar et al. reported an 18-year-old female with primary amenorrhea, absent uterus, vagina and right ovary as well as single ectopic grossly hydronephrotic kidney [65]. These cases of combined ovarian absence and Müllerian anomalies suggest an insult that occurs early in embryologic development. Such an insult accounts for both fallopian tube and OA. This supports existence of a congenital etiology of UOA, while acknowledging the implausibility of OA in the absence of ipsilateral renal findings.

Animal studies showed that ablation of genes involved in genital ridge development can lead to gonadal agenesis. *Lhx1*-, *Lhx9*-, *Emx2*-, *Nr5a1*-, and *Wt1*-null mice lack gonads completely, but agenesis extends to the kidneys and sometimes other organs, including the adrenal glands, due to their common embryological origin [5–9]. Ablation of additional factors has been shown to affect gonadal development. These genes include *Foxl2*, *Wnt4*, *Tcf21* (*Pod1)*, *Six1/4*, and members of the insulin/insulin-like growth factor family *Insr*, *Igf1r* and *Insrr* [66–70]. However, such deletions result in gonadal hypoplasia, dysgenesis, or sex reversal rather than agenesis. These findings show how the phenotypic presentation is directly associated with the time and tissue of expression of the gene being mutated or deleted. It is sometimes difficult to transpose animal studies to human cases. For example, *Wt1*and *Nr5a1*mutations in humans are associated with gonadal dysgenesis and primary ovarian insufficiency respectively, but do not impair early gonadal formation [71, 72]. However, despite possible species-specific genetic differences, genetically-caused gonadal agenesis would be unlikely to occur in isolation. In addition, loss-of-function of genes that are necessary for genital ridge development and ovarian formation would be unlikely to cause unilateral agenesis. In such an event, mechanisms including mosaicism or hypomorphic mutations should be considered. However, without genomic investigation and functional validation, these hypotheses are difficult to demonstrate.

### Imaging versus surgery

Our results suggest that imaging alone is insufficient to diagnose UOA. Of the case reports that noted results of ovarian imaging prior to confirmatory surgery, 28% (16/57) of imaging results were inconsistent with operative findings. These findings echo Case 1, where three ultrasounds reported normal bilateral ovaries prior to surgery. As UOA is relatively uncommon, some degree of expectancy bias may contribute to these inconstancies. Imaging is also limited by the skill of the technician obtaining the study, as well as the individual who interprets it. Ultrasound was the most common imaging modality reported, and the modality most prone to error. More sensitive modalities, such as MRI, were often conducted post-operatively to ensure that the ovary was not heterotopic/ectopic or missed during surgery. Arguably the most sensitive test for UOA was laparoscopy, as it incidentally revealed every instance of UOA that was missed on imaging. While laparoscopy is generally a safe operative procedure, it still carries risks inherent to surgical intervention, and is likely unnecessary in the absence of other indications. For patients pursuing infertility evaluation, laparoscopy may be useful. It gives patients definite knowledge of their anatomy and can be performed in conjunction with procedures aimed at the evaluation of tubal patency.

### Fertility

Questions of how UOA may affect fertility are of great importance to patients with the condition. As approximately 20% of our cases presented with fertility concerns, it may be tempting to infer a causal relationship. While fertility was a major cause of presentation, approximately 35% of our cases with ages ≥ 18 years had a history of pregnancy prior to their UOA diagnosis. Perhaps the most extreme example was an incidental UOA finding in a G11P11 woman with a history of 11 uncomplicated deliveries [73]. Another incidental UOA finding occurred during a cesarean delivery [74]. Interestingly, fertility was the focus of a prior review of 60 cases [75]. The study found UOA was not associated with adverse reproductive outcomes in the subset of women without other conditions that could decrease fertility (e.g. endometriosis, leiomyoma, uterine malformation). That is, comorbidities were likely responsible for sub/infertility rather than UOA itself. UOA patients with contralateral tube obstruction have had successful pregnancies following salpingostomy [76], or mucous plug removal during HSG [77]. Two other instances of contralateral tube obstruction were also noted [78, 79].

Another way to conceptualize fertility in UOA involves examining women with a surgically absent ovary. A metanalysis of 21 studies examining ovarian reserve status-post unilateral oophorectomy found decreased ovarian pool quantity, but not quality [80]. Despite a decreased response to induction during in vitro fertilization, the likelihood of a clinical pregnancy in women with one ovary was statistically comparable to women with two ovaries. Taken together, these findings suggest that infertility should not be presumed in UOA patients. In UOA patients without additional conditions that affect fertility the great majority were able to conceive, though some required assistive reproductive techniques. This group of patients roughly corresponds to our cases with suspected non-embryological UOA etiologies – as structural uterine anomalies were much less common in this group.

### Clinical practice

#### Recommendation

Upon suspicion for UOA, we suggest several practices to confirm the diagnosis. 1) Rule out ovarian ectopy. An ectopic ovary may be detected along the line of descent – the presence of bilaterally intact fallopian tubes may increase the suspicion for maldescent [81]. If a heterotopic location is confirmed, standard of care surveillance of the gonad is warranted [82]. 2) For suspected UOA with ipsilateral tubal abnormalities, providers may consider autoamputation. An autoamputated ovary may have implanted itself within the peritoneal cavity, and may be found within the omentum, intestinal serosa, or peritoneal wall [34, 35, 83]. Many prior reports of ovarian autoamputations have revealed free-floating masses in gravity-dependent areas, suggestive of calcified ovaries [64]. Documenting the presence and gross appearance of pelvic and abdominal organs in the operative findings of non-emergent laparoscopic procedure should be standard practice.

3) Upon ruling in UOA, preservation of the remaining ovary should be considered. Prior instances where the remaining ovary was removed due to pathology have resulted in surgical menopause [38]. Studies show that in cases where one ovary is removed, the remaining ovary becomes hypertrophied [84, 85]. Prophylactic oophoropexy may be considered as these patients have one remaining ovary, possible history of prior torsion, and compensatory hypertrophy that may increase the risk of torsion. In addition to locating the ovary, one should also attempt to locate any missing adnexa. Bowel strangulation and perforation caused by an ectopic tube has been previously reported [86]. 4) While most of our cases had patent contralateral tubes, a minority of patients demonstrated contralateral tubal obstruction – a possible contributor to presentations of infertility or subfertility. Due to these findings a HSG should be considered during infertility evaluation as part of planned diagnostic laparoscopy to optimize diagnostic yield. 5) Though non-embryological etiologies likely account for the majority of UOA cases, congenital causes should be ruled out in every patient with UOA. Possible renal agenesis or ectopy must be evaluated using the least invasive imaging modality. Renal ultrasound is a convenient initial modality as it avoids radiation exposure, and the kidneys are located retroperitoneally with unique sonographic appearance. 6) While genetic testing (microarray/sequencing) is not routinely beneficial, it may be warranted in patients with complex presentations (concurrent uterine or renal anomalies, syndromic phenotype, bilateral OA with low suspicion for torsion). Subsequent biochemical or functional validation of identified variants can be pursued. Lastly, ovarian cysts identified in utero that persist may increase risk of torsion and autoamputation [64]. This may account for some instances of UOA through the torsion etiology outlined above. As such, we recommend implementing postnatal surveillance protocols that have been previously described [87, 88].

We could not comment on the inheritance pattern of the OA as, with few exceptions, family history was rarely reported in the cases we reviewed. One case report noted their patient had a healthy monozygotic twin with no known gynecological pathology [29]. The maternal grandmother of our patient in Case 1 was thought to have had a unilateral oophorectomy for endometriosis, though documentation was not available. The minimal family history included in the reviewed case reports led the authors to consider non-embryological causes as the most likely etiology of the OA in our review. However, the possibility and extent of heritable genetic mutations contributing to embryological cases of UOA falls outside the scope of this review. In cases with suspected embryological etiology, genetic testing may be considered if presentations resemble characterized syndromes. Outside of these instances, the clinical utility of genetic testing is likely minimal.

### Strengths and limitations

In addition to documenting reported cases of UOA in-depth, our systematic review revealed a discrepancy in the literature regarding the semantics of “absence” and “agenesis” to describe patients with a solitary ovary. We also highlight the association between OA and Müllerian and renal anomalies, instances of which may denote true agenesis. We used a strict inclusion/exclusion criterion to ensure that our reported cases reflected surgically confirmed cases of UOA. By excluding articles without confirmation, we attempted to eliminate the possibility of false positive reports – in line with our finding that imaging may be insufficient to diagnose UOA.

Our study has several limitations that must be considered in light of our study design. First, we chose to exclude conference abstracts and non-English language publications. Conference abstracts, generally speaking, are less rigorous than peer-reviewed articles [89, 90]. Non-English language articles were excluded due to insufficient translation resources. Our study included data from case reports and series, which are study designs that inherently have a high potential for bias, despite overall good critical appraisal results. We were unable to secure a full text copy of one manuscript that may have included relevant cases (see Supplementary Table 2). Some older case reports predated the advent of karyotyping, raising the possibility that some patients with DSD are reported in our results. We attempted to minimize this possibility by excluding all cases with suspicion for DSD. Lastly, aside from clinical intuition, there is no reliable way of completely ensuring that our division of cases reports by suspected etiology was verifiably correct. This was somewhat limited by the information that case report authors chose to include. We believe our determinations of etiology are relevant to the discussion of absence versus agenesis, and still can inform clinical judgement. Taken together, these limitations suggest that the incidence of UOA is higher than our results would suggest.

## Conclusion

UOA is a heterogenous condition that is occasionally noted during imaging or surgical exploration. Further work up should be based upon full consideration of patients’ history, symptoms, and clinical goals. Careful evaluation of clinical history may help identifying torsion or autoamputation events. UOA found incidentally during surgery should be followed up with additional imaging to rule out heterotopy/ectopy, and fully assess abdomen and pelvis for any sign of pathology or abnormal findings. If the other ovary has been removed or is absent, laboratory assessment to assess ovarian reserve could be helpful as this would suggest an unidentified, functional ovary. If fertility is desired, intraoperative assessment of tubal patency should be considered to avoid the need for separate invasive procedures. Additionally, oophoropexy of the identified ovary should be considered to prevent torsion of a single ovary. In the case of unilateral oophorectomy, caution should be taken prior to removing the sole existing ovary due to the risk of surgical menopause. Genetic analysis is not routinely beneficial in establishing a differential unless OA presents in association with congenital urogenital anomalies or true agenesis is suspected. In this case, biochemical or functional validation of the identified variants should be performed following genetic analysis. Overall, UOA does not seem to represent an independent risk factor for infertility or primary ovarian insufficiency. However, more data is required to definitively address these risks, and patients should be counseled accordingly. While true agenesis likely occurs early in embryogenesis, the possibility of predisposing heritable or environmental factors, as well as genetic associations, remains unknown. This review adds to the existing knowledge on OA and will provide guidance to clinicians, thus improving clinical care for patients with ovarian absence.

## Supplementary Information

**Additional file 1:** **Supplementary Table 1. **Reporting Guideline Checklists. **Supplementary Table 2.** Search Strategies & Additional Methodology. **Supplementary Table 3.** Table of Included Studies. **Supplementary Table 4. **Excluded Studies Table with reasons for Exclusion. **Supplementary Table 5. **JBI Critical Appraisal Checklist for Case Reports. **Figure S1.** Critical appraisal of included studies.

## Data Availability

A copy of the raw data can be requested from the corresponding author.
