# Supplementary material for: Ovarian absence: a systematic literature review and case series report
Source: J Ovarian Res. 2023 Jan 16;16:13. doi: 10.1186/s13048-022-01090-1 (PMC9841619; doi:10.1186/s13048-022-01090-1)
Supplement: Supplementary file 1 — Additional file 1: Supplementary Table 1. Reporting Guideline Checklists. Supplementary Table 2. Search Strategies & Additional Methodology. Supplementary Table 3. Table of Included Studies. Supplementary Table 4. Excluded Studies Table with reasons for Exclusion. Supplementary Table 5. JBI Critical Appraisal Checklist for Case Reports. Figure S1. Critical appraisal of included studies. [file 13048_2022_1090_MOESM1_ESM.docx]

**Supplementary Materials**

Manuscript: Ovarian Absence: A Systematic Literature Review and Case Series Report

**Supplementary Table 1: Reporting Guideline Checklists**

PRISMA 2020 Main Checklist

| **Topic** | **No.** | **Item** | **Location where item is reported** |
| --- | --- | --- | --- |
| **TITLE** |  |  |  |
| **Title** | 1 | Identify the report as a systematic review. | Cover Page |
| **ABSTRACT** |  |  |  |
| **Abstract** | 2 | See the PRISMA 2020 for Abstracts checklist | Suppl. 1 |
| **INTRODUCTION** |  |  |  |
| **Rationale** | 3 | Describe the rationale for the review in the context of existing knowledge. | LN 97-108. 144-163 |
| **Objectives** | 4 | Provide an explicit statement of the objective(s) or question(s) the review addresses. | LN 159-163 |
| **METHODS** |  |  |  |
| **Eligibility criteria** | 5 | Specify the inclusion and exclusion criteria for the review and how studies were grouped for the syntheses. | LN 185-194 |
| **Information sources** | 6 | Specify all databases, registers, websites, organisations, reference lists and other sources searched or consulted to identify studies. Specify the date when each source was last searched or consulted. | LN 170-183 |
| **Search strategy** | 7 | Present the full search strategies for all databases, registers and websites, including any filters and limits used. | LN 173-175 & Suppl. 2 |
| **Selection process** | 8 | Specify the methods used to decide whether a study met the inclusion criteria of the review, including how many reviewers screened each record and each report retrieved, whether they worked independently, and if applicable, details of automation tools used in the process. | LN 185-195 |
| **Data collection process** | 9 | Specify the methods used to collect data from reports, including how many reviewers collected data from each report, whether they worked independently, any processes for obtaining or confirming data from study investigators, and if applicable, details of automation tools used in the process. | LN 196-199 |
| **Data items** | 10a | List and define all outcomes for which data were sought. Specify whether all results that were compatible with each outcome domain in each study were sought (e.g. for all measures, time points, analyses), and if not, the methods used to decide which results to collect. | LN 196-199 |
|  | 10b | List and define all other variables for which data were sought (e.g. participant and intervention characteristics, funding sources). Describe any assumptions made about any missing or unclear information. | LN 196-199 |
| **Study risk of bias assessment** | 11 | Specify the methods used to assess risk of bias in the included studies, including details of the tool(s) used, how many reviewers assessed each study and whether they worked independently, and if applicable, details of automation tools used in the process. | LN 201-210 |
| **Effect measures** | 12 | Specify for each outcome the effect measure(s) (e.g. risk ratio, mean difference) used in the synthesis or presentation of results. | - |
| **Synthesis methods** | 13a | Describe the processes used to decide which studies were eligible for each synthesis (e.g. tabulating the study intervention characteristics and comparing against the planned groups for each synthesis (item 5)). | LN 212-220 |
|  | 13b | Describe any methods required to prepare the data for presentation or synthesis, such as handling of missing summary statistics, or data conversions. | LN 212-220 |
|  | 13c | Describe any methods used to tabulate or visually display results of individual studies and syntheses. | LN 212-220 |
|  | 13d | Describe any methods used to synthesize results and provide a rationale for the choice(s). If meta-analysis was performed, describe the model(s), method(s) to identify the presence and extent of statistical heterogeneity, and software package(s) used. | LN 212-220 |
|  | 13e | Describe any methods used to explore possible causes of heterogeneity among study results (e.g. subgroup analysis, meta-regression). | LN 212-220 |
|  | 13f | Describe any sensitivity analyses conducted to assess robustness of the synthesized results. | LN 212-220 |
| **Reporting bias assessment** | 14 | Describe any methods used to assess risk of bias due to missing results in a synthesis (arising from reporting biases). | Suppl. 3 |
| **Certainty assessment** | 15 | Describe any methods used to assess certainty (or confidence) in the body of evidence for an outcome. | - |
| **RESULTS** |  |  |  |
| **Study selection** | 16a | Describe the results of the search and selection process, from the number of records identified in the search to the number of studies included in the review, ideally using a flow diagram. | LN 295-302, Figure 2 |
|  | 16b | Cite studies that might appear to meet the inclusion criteria, but which were excluded, and explain why they were excluded. | Suppl. 4 |
| **Study characteristics** | 17 | Cite each included study and present its characteristics. | LN 304-312 |
| **Risk of bias in studies** | 18 | Present assessments of risk of bias for each included study. | LN 308-313 Suppl. 3 |
| **Results of individual studies** | 19 | For all outcomes, present, for each study: (a) summary statistics for each group (where appropriate) and (b) an effect estimate and its precision (e.g. confidence/credible interval), ideally using structured tables or plots. | Table 1 |
| **Results of syntheses** | 20a | For each synthesis, briefly summarise the characteristics and risk of bias among contributing studies. | Suppl. 3, 208-213 |
|  | 20b | Present results of all statistical syntheses conducted. If meta-analysis was done, present for each the summary estimate and its precision (e.g. confidence/credible interval) and measures of statistical heterogeneity. If comparing groups, describe the direction of the effect. | LN 376-461 |
|  | 20c | Present results of all investigations of possible causes of heterogeneity among study results. | - |
|  | 20d | Present results of all sensitivity analyses conducted to assess the robustness of the synthesized results. | LN 376-461 |
| **Reporting biases** | 21 | Present assessments of risk of bias due to missing results (arising from reporting biases) for each synthesis assessed. | - |
| **Certainty of evidence** | 22 | Present assessments of certainty (or confidence) in the body of evidence for each outcome assessed. | - |
| **DISCUSSION** |  |  |  |
| **Discussion** | 23a | Provide a general interpretation of the results in the context of other evidence. | LN 463-684 |
|  | 23b | Discuss any limitations of the evidence included in the review. | LN 674-684 |
|  | 23c | Discuss any limitations of the review processes used. | LN 698-712 |
|  | 23d | Discuss implications of the results for practice, policy, and future research. | LN 714-735 |
| **OTHER INFORMATION** |  |  |  |
| **Registration and protocol** | 24a | Provide registration information for the review, including register name and registration number, or state that the review was not registered. | LN 739 |
|  | 24b | Indicate where the review protocol can be accessed, or state that a protocol was not prepared. | LN 739 |
|  | 24c | Describe and explain any amendments to information provided at registration or in the protocol. | - |
| **Support** | 25 | Describe sources of financial or non-financial support for the review, and the role of the funders or sponsors in the review. | LN 738 |
| **Competing interests** | 26 | Declare any competing interests of review authors. | LN 736-737 |
| **Availability of data, code and other materials** | 27 | Report which of the following are publicly available and where they can be found: template data collection forms; data extracted from included studies; data used for all analyses; analytic code; any other materials used in the review. | Available upon request |

From: Page MJ, McKenzie JE, Bossuyt PM, Boutron I, Hoffmann TC, Mulrow CD, et al. The PRISMA 2020 statement: an updated guideline for reporting systematic reviews. MetaArXiv. 2020, September 14. DOI: 10.31222/osf.io/v7gm2. For more information, visit: www.prisma-statement.org

# PRISMA Abstract Checklist

| **Topic** | **No.** | **Item** | **Reported?** |
| --- | --- | --- | --- |
| **TITLE** |  |  |  |
| **Title** | 1 | Identify the report as a systematic review. | Yes |
| **BACKGROUND** |  |  |  |
| **Objectives** | 2 | Provide an explicit statement of the main objective(s) or question(s) the review addresses. | Yes |
| **METHODS** |  |  |  |
| **Eligibility criteria** | 3 | Specify the inclusion and exclusion criteria for the review. | Yes |
| **Information sources** | 4 | Specify the information sources (e.g. databases, registers) used to identify studies and the date when each was last searched. | Yes |
| **Risk of bias** | 5 | Specify the methods used to assess risk of bias in the included studies. | No |
| **Synthesis of results** | 6 | Specify the methods used to present and synthesize results. | Yes |
| **RESULTS** |  |  |  |
| **Included studies** | 7 | Give the total number of included studies and participants and summarise relevant characteristics of studies. | Yes |
| **Synthesis of results** | 8 | Present results for main outcomes, preferably indicating the number of included studies and participants for each. If meta-analysis was done, report the summary estimate and confidence/credible interval. If comparing groups, indicate the direction of the effect (i.e. which group is favoured). | Yes |
| **DISCUSSION** |  |  |  |
| **Limitations of evidence** | 9 | Provide a brief summary of the limitations of the evidence included in the review (e.g. study risk of bias, inconsistency and imprecision). | Yes |
| **Interpretation** | 10 | Provide a general interpretation of the results and important implications. | Yes |
| **OTHER** |  |  |  |
| **Funding** | 11 | Specify the primary source of funding for the review. | Yes |
| **Registration** | 12 | Provide the register name and registration number. | Yes |

The citation for the Synthesis Without Meta-analysis explanation and elaboration article is: Campbell M, McKenzie JE, Sowden A, Katikireddi SV, Brennan SE, Ellis S, Hartmann-Boyce J, Ryan R, Shepperd S, Thomas J, Welch V, Thomson H. Synthesis without meta-analysis (SWiM) in systematic reviews: reporting guideline BMJ 2020;368:l6890 <http://dx.doi.org/10.1136/bmj.l6890>

| **SWiM is intended to complement and be used as an extension to PRISMA** | | | |
| --- | --- | --- | --- |
| **SWiM reporting item** | **Item description** | **Page in manuscript where item is reported** | **Other*** |
| *Methods* | | | |
| **1** Grouping studies for synthesis | 1a) Provide a description of, and rationale for, the groups used in the synthesis (e.g., groupings of populations, interventions, outcomes, study design) | 5-7 |  |
|  | 1b) Detail and provide rationale for any changes made subsequent to the protocol in the groups used in the synthesis | N/A |  |
| **2** Describe the standardised metric and transformation methods used | Describe the standardised metric for each outcome. Explain why the metric(s) was chosen, and describe any methods used to transform the intervention effects, as reported in the study, to the standardised metric, citing any methodological guidance consulted | 5-7 |  |
| **3** Describe the synthesis methods | Describe and justify the methods used to synthesise the effects for each outcome when it was not possible to undertake a meta-analysis of effect estimates | 5-7 |  |
| **4** Criteria used to prioritise results for summary and synthesis | Where applicable, provide the criteria used, with supporting justification, to select the particular studies, or a particular study, for the main synthesis or to draw conclusions from the synthesis (e.g., based on study design, risk of bias assessments, directness in relation to the review question) | 5-7 |  |

| **SWiM reporting item** | **Item description** | **Page in manuscript where item is reported** | **Other*** |
| --- | --- | --- | --- |
| **5** Investigation of heterogeneity in reported effects | State the method(s) used to examine heterogeneity in reported effects when it was not possible to undertake a meta-analysis of effect estimates and its extensions to investigate heterogeneity | 5-7 |  |
| **6** Certainty of evidence | Describe the methods used to assess certainty of the synthesis findings | 5-7 |  |
| **7** Data presentation methods | Describe the graphical and tabular methods used to present the effects (e.g., tables, forest plots, harvest plots).  Specify key study characteristics (e.g., study design, risk of bias) used to order the studies, in the text and any tables or graphs, clearly referencing the studies included | 5-7 |  |
| *Results* | | | |
| **8** Reporting results | For each comparison and outcome, provide a description of the synthesised findings, and the certainty of the findings. Describe the result in language that is consistent with the question the synthesis addresses, and indicate which studies contribute to the synthesis | 14-19 |  |
| *Discussion* |  |  |  |
| **9** Limitations of the synthesis | Report the limitations of the synthesis methods used and/or the groupings used in the synthesis, and how these affect the conclusions that can be drawn in relation to the original review question | 27-28 |  |

PRISMA=Preferred Reporting Items for Systematic Reviews and Meta-Analyses.

*If the information is not provided in the systematic review, give details of where this information is available (e.g., protocol, other published papers (provide citation details), or website (provide the URL)).

** CARE Checklist (2013) of information to include when writing a case report**

| **Topic** | **Item** | **Checklist item description** | **Reported on Page** |
| --- | --- | --- | --- |
| **Title** | **1** | The words “case report” should be in the title along with the area of focus . . . . . . . . . . . . . . . . . . . . . . . . . . . . . . . . . . . | **1** |
| **Key Words** | **2** | 2 to 5 key words that identify areas covered in this case report. . . . . . . . . . . . . . . . . . . . . . . . . . . . . . . . . . . . . . . . . . . . . . . . . . . . . . . | **2** |
| **Abstract** | **3a** | Introduction—What is unique about this case? What does it add to the medical literature? . . . . . . | **2** |
|  | **3b** | The main symptoms of the patient and the important clinical findings . . . . . . . . . . . . . . . . . . . . . . . . | **2** |
|  | **3c** | The main diagnoses, therapeutics interventions, and outcomes . . . . . . . . . . . . . . . . . . . . . . . . . . . . | **2** |
|  | **3d** | Conclusion—What are the main “take-away” lessons from this case? . . . . . . . . . . . . . . . . . . . . . . | **2** |
| **Introduction** | **4** | One or two paragraphs summarizing why this case is unique with references . . . . . . . | **3-5** |
| **Patient Information** | **5a** | De-identified demographic information and other patient specific information . . . . . . . . . | **7-8, 10-11** |
|  | **5b** | Main concerns and symptoms of the patient . . . . . . . . . . . . . . . . . . . . . . . . . . . . . . . . . . . . . . . . | **7-8, 10-11** |
|  | **5c** | Medical, family, and psychosocial history including relevant genetic information (also see timeline). . . | **7-11** |
|  | **5d** | Relevant past interventions and their outcomes . . . . . . . . . . . . . . . . . . . . . . . . . . . . . . . . . . . . . | **7-11** |
| **Clinical Findings** | **6** | Describe the relevant physical examination (PE) and other significant clinical findings. | **7-11** |
| **Timeline** | **7** | Important information from the patient’s history organized as a timeline . . . . . . . . . . . . | **7-11** |
| **Diagnostic Assessment** | **8a** | Diagnostic methods (such as PE, laboratory testing, imaging, surveys). . . . . . . . . . . . . . . | **7-11** |
|  | **8b** | Diagnostic challenges (such as access, financial, or cultural) . . . . . . . . . . . . . . . . . . . . . . . . | **7-11** |
|  | **8c** | Diagnostic reasoning including other diagnoses considered . . . . . . . . . . . . . . . . . . . . . . . . . | **7-11** |
|  | **8d** | Prognostic characteristics (such as staging in oncology) where applicable . . . . . . . . . . . . | **7-11** |
| **Therapeutic Intervention** | **9a** | Types of intervention (such as pharmacologic, surgical, preventive, self-care) . . . . . . . . . . . . . . . . | **7-11** |
|  | **9b** | Administration of intervention (such as dosage, strength, duration) . . . . . . . . . . . . . . . . . . . . . . . . . | **7-11** |
|  | **9c** | Changes in intervention (with rationale) . . . . . . . . . . . . . . . . . . . . . . . . . . . . . . . . . . . . . . . . . . . . . . . . | **-** |
| **Follow-up and**  **Outcomes** | **10a** | Clinician and patient-assessed outcomes (when appropriate) . . . . . . . . . . . . . . . . .. . . . . . . . . . . . . | **7-11** |
|  | **10b** | Important follow-up diagnostic and other test results . . . . . . . . . . . . . . . . . . . . . . . . . . . . . . . . . . . . . | **7-11** |
|  | **10c** | Intervention adherence and tolerability (How was this assessed?) . . . . . . . . . . . . . . . . . . . . . . . . . . | **7-11** |
|  | **10d** | Adverse and unanticipated events . . . . . . . . . . . . . . . . . . . . . . . . . . . . . . . . . . . . . . . . . . . . . . . . . . . . | **7-11** |
| **Discussion** | **11a** | Discussion of the strengths and limitations in your approach to this case . . . . . . . . . . . . . . . . . . . . . | **27-28** |
|  | **11b** | Discussion of the relevant medical literature. . . . . . . . . . . . . . . . . . . . . . . . . . . . . . . . . . . . . . . . . . . . . | **19-25** |
|  | **11c** | The rationale for conclusions (including assessment of possible causes) . . . . . . . . . . . . . . . . . . . . | **20-24** |
|  | **11d** | The primary “take-away” lessons of this case report . . . . . . . . . . . . . . . . . . . . . . . . . . .. . . . . . . . . . . | **28-29** |
| **Patient Perspective** | **12** | When appropriate the patient should share their perspective on the treatments they received . . | **-** |
| **Informed Consent** | **13** | Did the patient give informed consent? Please provide if requested . . . . . . . . . . . . . . . . . . . . . . . . . | **Yes** |
|  |  |  |  |

Supplementary Table 2: Search Strategies & Additional Methodology

**Search for Ovid Embase**

1. exp agenesis/ or ectopic organ/

2. exp ovary/ or exp ovary disease/

3. 1 and 2

4. ((lack or absence or absent or insufficien* or undescend* or abnormal or dysgenes* or aplasia or agenesis or ageneses or agenesia or hypoplas* or heterotop* or ectopic* or supernumerar* ) adj3 (primordial tissue* or primordium or ovaries or ovary or ovarian tissue* or oophor* or tubo-ovar*)).tw,kw.

5. (no adj1 (primordial tissue* or primordium or ovaries or ovary or ovarian tissue* or oophor* or tubo-ovar*)).tw,kw.

6. (UOA and (ovaries or ovary or ovarian tissue* or tubo-ovar* or oophor*)).ti,ab.

7. ((ovar* or adnexal) adj3 torsion* adj3 (in utero* or neonatal)).tw,kw.

8. OR/3-7

9. gondal agenesis/

10. (gonad* adj3 (lack or absence or absent or undescended or abnormal or aplasia or agenesis or agenesia or ageneses or hypoplas* or heterotop* or ectopic*)).tw,kw.

11. 9 or 10

12. (women or woman or female* or girl*).tw,kw.

13. exp female/

14. 12 or 13

15. 11 and 14

16. 8 or 15

17. exp animal/

18. exp animal/ and exp human/

19. 17 not 18

20. 16 not 19

**Search for Ovid Medline**

1. abnormalities.fs.

2. exp ovary/ or exp ovarian diseases/

3. 1 and 2

4. ((lack or absence or absent or insufficien* or undescend* or abnormal or dysgenes* or aplasia or agenesis or ageneses or agenesia or hypoplas* or heterotop* or ectopic* or supernumerar* ) adj3 (primordial tissue* or primordium or ovaries or ovary or ovarian tissue* or oophor* or tubo-ovar*)).tw,kf.

5. (no adj1 (primordial tissue* or primordium or ovaries or ovary or oophor* or tubo-ovar*)).tw,kf.

6. (UOA and (ovaries or ovary or ovarian tissue* or tubo-ovar* or oophor*)).ti,ab.

7. ((ovar* or adnexal) adj3 torsion* adj3 (in utero* or neonatal)).tw,kf.

8. OR/3-7

9. gonadal dysgenesis, 46,XX / or gonadal dysgenesis/

10. (gonad* adj3 (lack or absence or absent or undescended or abnormal or aplasia or agenesis or agenesia or ageneses or hypoplas* or heterotop* or ectopic*)).tw,kf.

11. 9 or 10

12. (women or woman or female* or girl*).tw,kf.

13. exp female/

14. 12 or 13

15. 11 and 14

16. 8 or 15

17. exp animals/

18. exp animals/ and exp humans/

19. 17 not 18

20. 16 not 19

**Search for Web of Science Core Collection**

#1 TS=((lack or absence or absent or insufficien* or undescend* or abnormal or dysgenes* or aplasia or agenesis or ageneses or agenesia or hypoplas* or heterotop* or ectopic* or supernumerar* ) near/3 ("primordial tissue*" or primordium or ovaries or ovary or "ovarian tissue*" or oophor* or tubo-ovar*) ) or TS=(UOA and (ovaries or ovary or "ovarian tissue*" or tubo-ovar* or oophor*) ) OR TS=((ovaries or ovary or "ovarian tissue*" or adnexal) near/3 torsion* near/3 ("in utero*" or neonatal) ) OR TS=(no near/1 ("primordial tissue*" or primordium or ovaries or ovary or "ovarian tissue" or oophor* or tubo-ovar*))

#2 TS=(gonad* near/3 (lack or absence or absent or undescended or abnormal or aplasia or agenesis or agenesia or ageneses or hypoplas* or heterotop* or ectopic*)) AND TS=(women or woman or female* or girl*)

#1 and #2

**Search for Scopus**

#1 TITLE-ABS-KEY((lack or absence or absent or insufficien* or undescend* or abnormal or dysgenes* or aplasia or agenesis or ageneses or agenesia or hypoplas* or heterotop* or ectopic* or supernumerar* ) W/3 ("primordial tissue*" or primordium or ovaries or ovary or "ovarian tissue*" or oophor* or tubo-ovar*) ) or TITLE-ABS-KEY(UOA and (ovaries or ovary or "ovarian tissue*" or tubo-ovar* or oophor*) ) OR TITLE-ABS-KEY((ovaries or ovary or "ovarian tissue*" or adnexal) W/3 torsion* W/3 ("in utero*" or neonatal) ) OR TITLE-ABS-KEY (no W/1 ("primordial tissue*" or primordium or ovaries or ovary or "ovarian tissue" or oophor* or tubo-ovar*))

#2 TITLE-ABS-KEY (gonad* W/3 (lack or absence or absent or undescended or abnormal or aplasia or agenesis or agenesia or ageneses or hypoplas* or heterotop* or ectopic*)) AND TITLE-ABS-KEY (women or woman or female* or girl*)

#1 and #2

**Search for Google Scholar**

ovarian agenesis

**Search for ClinicalTrials.gov**

Condition or disease Search: ovarian agenesis

**Search for Pubmed**

Absent ovary[Title/Abstract] OR insufficien* ovary[Title/Abstract] OR undescend* ovary[Title/Abstract] OR abnormal ovary[Title/Abstract] OR dysgenes* ovary[Title/Abstract] OR aplasia ovary[Title/Abstract] OR ovary agenesis[Title/Abstract] OR ovary ageneses[Title/Abstract] OR ovary agenesia[Title/Abstract] OR ovary hypoplas*[Title/Abstract] OR ovary heterotop*[Title/Abstract] OR ectopic* ovary[Title/Abstract] OR ovary supernumerar*[Title/Abstract] OR absent ovaries[Title/Abstract] OR insufficien* ovaries[Title/Abstract] OR undescend* ovaries[Title/Abstract] OR abnormal ovaries[Title/Abstract] OR ovarian dysgenes*[Title/Abstract] OR ovaries aplasia[Title/Abstract] OR ovaries agenesis[Title/Abstract] OR ovaries ageneses[Title/Abstract] OR ovaries agenesia[Title/Abstract] OR ovaries hypoplas*[Title/Abstract] OR heterotop* ovaries[Title/Abstract] OR ectopic* ovaries[Title/Abstract] OR supernumerar* ovaries[Title/Abstract] OR ovarian ageneses[Title/Abstract] OR ovarian agenesia[Title/Abstract] OR ovarian agenesis[Title/Abstract] OR ovarian hypoplas*[Title/Abstract] OR ovarian supernumerar*[Title/Abstract]

**Search for Cochrane Library**

#1 ((lack or absence or absent or insufficien* or undescend* or abnormal or dysgenes* or aplasia or agenesis or ageneses or agenesia or hypoplas* or heterotop* or ectopic* or supernumerar* ) near/3 ("primordial tissue*" or primordium or ovaries or ovary or "ovarian tissue*" or oophor* or tubo-ovar*)):ti,ab or (UOA and (ovaries or ovary or "ovarian tissue*" or tubo-ovar* or oophor*)):ti,ab OR ((ovaries or ovary or "ovarian tissue*" or adnexal) near/3 torsion* near/3 ("in utero*" or neonatal)):ti,ab OR (no near/1 ("primordial tissue*" or primordium or ovaries or ovary or "ovarian tissue" or oophor* or tubo-ovar*)):ti,ab

#2 (gonad* near/3 (lack or absence or absent or undescended or abnormal or aplasia or agenesis or agenesia or ageneses or hypoplas* or heterotop* or ectopic*)):ti,ab AND (women or woman or female* or girl*):ti,ab

#3 #1 or #2

**Methods of Full-Text Retrieval :**

Citation that could not be retrieved:

- Stuti, T.; Suchitra, J.; Surinder, S.; Pragya, Y. An incidental finding of unicornuate uterus with unilateral ovarian agenesis and ipsilateral twining of fallopian tubes during cesarean. JARMS 2015;7(1):11-13

One paper identified from the database search that we were unable to obtain the full-text manuscript of either of the papers. This paper was requested through interlibrary loan three different times between 2020-2021. It should be noted that the National Library of Medicine’s physical collection was not being scanned due to the COVID-19 pandemic and library closure. When unable to obtain an interlibrary loan for the material, the team then sent out requests to several medical library listservs, the National Library of India, and several authors of that cited the paper. We received one email in response to these requests with the incorrect manuscript attached. After clarification, it was determined that they also did not have access to the correct manuscript.

| Supplementary Table 3: Table of Included Studies | | | | | | | | |
| --- | --- | --- | --- | --- | --- | --- | --- | --- |
| **Reference Information** | **Patient Age**  **(in years)** | **Presentation/ Procedure** | **Ovarian Agenesis -Affected Side** | **Imaging** | **Other Reproductive Tract Findings** | **Kidney Findings** | **Study Design** | **Oxford Scale of Evidence** |
| Abargel et al. 2000^1^ | 9 | abdominal/ pelvic pain | left | ultrasound | absent adnexa with partial tube | Not specified | Case Report | Level 5 |
| Alexander 1947^2^ | 23 | abdominal/ pelvic pain | left | - |  | Abnormal | Case Report | Level 5 |
| Ali et al. 1979^3^ | 22 | abdominal/ pelvic pain | right | ultrasound,  X-ray | right distal tube absent | Normal | Case Report | Level 5 |
| Alrabeeah et al. 1988^4^ | 4 days | evaluation of mass | right | ultrasound,  X-ray |  | Normal | Case Series | Level 4 |
| Alvir et al. 2013^5^ | 38 | sterilization | left | ultrasound | left tube and round absent, unicornuate uterus | Abnormal | Case Report | Level 5 |
| Aslam et al. 1995^6^ | 11 months | evaluation of mass | right | ultrasound | blind ending right tube, cyst found in right abdomen | Not specified | Case Report | Level 5 |
| Awad et al. 2018^7^ | 36 | sterilization | right | - | IP lig absent, tube was attenuated, elongated, and adherent to bowel | Not specified | Case Report | Level 5 |
| Barsky et al. 2015^8^ | 13 | amenorrhea | left | ultrasound, CT scan | clubbed left tube without fimbria | Normal | Case Report | Level 5 |
|  | 31 | abdominal/ pelvic pain | right | MRI | right tube absent,  large left ovarian mass | Normal |  |  |
| Bates et al. 1982^9^ | 24 | infertility/  subfertility | right | HSG or chromotubation | left and right fallopian tubes extended 1.4 cm beyond cornu and terminated blindly | Not specified | Case Report | Level 5 |
| Bay et al. 2014^10^ | 48 | malignancy | right | ultrasound, CT scan |  | Not specified | Case Report | Level 5 |
| Ben-Nun et al. 1988^11^ | 34 | infertility/  subfertility | left | HSG or chromotubation | left ovary absent, right ovary slightly enlarged | Not specified | Case Report | Level 5 |
| Bilij-Erski et al. 2019^12^ | 34 | infertility/  subfertility | right | - | right ovary and tube absent | Normal | Case Report | Level 5 |
| Blumberg et al. 1996^13^ | 10 months | Abdominal mass | right | ultrasound | right ovary and distal tube absent | Normal | Case Report | Level 5 |
| Bousfiha et al. 2010^14^ | 19 | amenorrhea | bilateral | ultrasound, MRI |  | Normal | Case Report | Level 5 |
| Bradley et al. 1980^15^ | 50 | endometrial hyperplasia | left | - | left tube and ovary absent | Abnormal | Case Report | Level 5 |
| Bugmann et al. 2001^16^ | 14 | abdominal/ pelvic pain | left | ultrasound,  X-ray | left tube ectopic, absent uterus | Normal | Case Report | Level 5 |
| Burge 1958^17^ | 19 | abdominal/ pelvic pain | left | - |  | Not specified | Case Report | Level 5 |
| Castellani et al. 2013^18^ | 2.5 months | evaluation of mass | left | ultrasound |  | Not specified | Case Report | Level 5 |
| Chan et al. 1987^19^ | 25 | infertility/  subfertility | bilateral | - | absent tubes | Normal | Case Report | Level 5 |
| Chen et al. 2014^20^ | 26 | infertility/  subfertility | left | ultrasound |  | Normal | Case Report | Level 5 |
| Choundhary et al. 2017^21^ | 19 | abdominal/ pelvic pain | right | ultrasound | right tube absent | Normal | Case Series | Level 4 |
|  | 26 | infertility/  subfertility | left | ultrasound, HSG or chromotubation | partial left tube | Normal |  |  |
| Cucinella et al. 2013^22^ | 18 | abdominal/ pelvic pain | left | ultrasound | left ovary, tube, and broad and round ligaments absent | Normal | Case Report | Level 5 |
| Currarino et al. 1989^23^ | 2 | abdominal/ pelvic pain | right | X-ray | right tube absent | Not specified | Case Series | Level 4 |
|  | 1.17 | abdominal/ pelvic pain | right | X-ray | normal uterus, but dislocated to left | Not specified |  |  |
| Dannreuther 1923^24^ | 25 | abdominal/ pelvic pain | left | - | Left tube, broad, and round ligaments absent | Abnormal | Case Report | Level 5 |
| Dare et al. 1989^25^ | 45 | AUB | left | - |  | Not specified | Case Report | Level 5 |
| De et al. 2019^26^ | 13 | abdominal/ pelvic pain | left | ultrasound, MRI |  | Abnormal | Case Report | Level 5 |
| Dede et al. 2008^27^ | 18 | amenorrhea | bilateral | ultrasound | fibroids | Normal | Case Report | Level 5 |
| Demir et al. 2007^28^ | 31 | C/S | left | ultrasound | left tube or round ligaments absent, unicornuate uterus | Normal | Case Report | Level 5 |
| Dueck et al. 2001^29^ | 2 months | evaluation of mass | left | ultrasound | unicornuate uterus, hypoplastic/rudimentary uterus, rudimentary left tube | Not specified | Case Report | Level 5 |
| Durous et al. 2019^30^ | 26 | evaluation of mass | right | ultrasound, CT scan, MRI | blindly ending right tube and IP ligment, free floating pelvic mass in posterior cul de sac | Not specified | Case Report | Level 5 |
| Eda et al. 2012^31^ | 83 | autopsy | left | - | partial tubal absence | Not specified | Case Report | Level 5 |
| Eustace 1992^32^ | 24 | infertility/  subfertility | right | HSG or chromotubation | blindly ending tubes bilaterally | Not specified | Case Report | Level 5 |
|  | 35 | sterilization | right |  | blindly ending right tube | Not specified |  |  |
| Fletcher et al. 1988^33^ | 2 | evaluation of mass | bilateral | ultrasound, CT scan |  | Normal | Case Report | Level 5 |
| Forceseneanu et al. 2013^34^ | 9 | abdominal/ pelvic pain | left | ultrasound, CT scan, | Blindly ending left tube and ligament | Not specified | Case Series | Level 4 |
|  | 1.42 | evaluation of mass | left | CT scan | left tube ended | Normal |  |  |
| Galli et al. 2017^35^ | 21 | amenorrhea | left | ultrasound | left tube absent | Normal | Case Report | Level 5 |
| Guthrie et al. 1909^36^ | 30 | abdominal/ pelvic pain | left | - | left tube and broad ligament absent | Abnormal | Case Report | Level 5 |
| Gutierrez 1933^37^ | 26 | congenital abnormality | left | - | left tube absent, hypoplastic cervix | Abnormal | Case Report | Level 5 |
| Haydardedeoglu et al. 2006^38^ | 48 | menometrorrhagia | left | ultrasound | Left tube absent | Abnormal | Case Report | Level 5 |
| Kennedy et al. 1981^39^ | 1.42 | abdominal/ pelvic pain | left | CT scan | blindly ending left tube, fibroids, unspecified vaginal/uterine tumor, rudimentary labia majora, absent labia minora, absent vagina | Normal | Case Series | Level 4 |
|  | .42 | evaluation of mass | right | X-ray, urogram | right tube rudimentary, left sided underdevelopment | Normal |  |  |
|  | 9 | evaluation of mass | right | Urogram, barium enema |  | Normal |  |  |
|  | 2 weeks | evaluation of mass | right | Urogram, barium enema | tube absent | Normal |  |  |
| Kent 1956^40^ | 23 | ectopic, endometrial hyperplasia | left | - | absent tube with hemorrhage at uterine side where tube should have been | Normal | Case Report | Level 5 |
| Koh 1974^41^ | 34 | sterilization | left | - | Left tube, round and broad ligaments absent | Normal | Case Report | Level 5 |
| Kriplani et al. 1995^42^ | 36 | infertility/  subfertility | bilateral | - | blindly ending tubes bilaterally | Normal | Case Report | Level 5 |
| Kumar et al. 2007^43^ | 18 | amenorrhea | right | ultrasound, MRI | uterus absent | Abnormal | Case Report | Level 5 |
| Kurcz et al. 1948^44^ | 30 | AUB | right | - | narrow introitus, dry vagina, small uterus, small right tube | Abnormal | Case Report | Level 5 |
| Kusaka et al. 2007^45^ | 24 | evaluation of mass | left | ultrasound, MRI | no identifiable left tube, absent uterus, prepubertal vagina ending in blind pouch | Normal | Case Report | Level 5 |
| Lashgari 1975^46^ | 33 | sterilization | left | X-ray | left tube absent | Normal | Case Report | Level 5 |
| Lee et al. 2016^47^ | 77 | abdominal/ pelvic pain | right | ultrasound, CT scan | right tube absent | Normal | Case Report | Level 5 |
| Liu et al. 2013^48^ | 23 | abdominal/ pelvic pain | right | ultrasound | right tube, broad and round ligaments absent | Abnormal | Case Series | Level 4 |
| Loo et al. 2020^49^ | 26 | abdominal/ pelvic pain | left | ultrasound | no left tube, unicornuate uterus | Normal | Case Report | Level 5 |
| Luvero et al. 2016^50^ | 30 | infertility/subfertility | right | ultrasound, MRI | right tube absent | Normal | Case Report | Level 5 |
| Mamah et al. 2022 | 36 | amenorrhea | left | ultrasound | left tube absent | Abnormal | Case Report | Level 5 |
| Mecklenburg et al. 1974^51^ | 1 day | amenorrhea | left | - | left tube absent, mature cystic teramoma | Abnormal | Case Report | Level 5 |
| Metoki et al. 1986^52^ | 4 months | congenital abnormality | left | - |  | Abnormal | Case Report | Level 5 |
| Mishell 1938^53^ | 24 | abdominal/ pelvic pain | right | - | uterus absent | Not specified | Case Report | Level 5 |
| Morao et al. 2017^54^ | Not Specified | congenital abnormality | right | ultrasound | right left and uterus absent | Abnormal | Case Report | Level 5 |
| Muppala et al. 2008^55^ | 22 | AUB | right | - | right tube and broad ligaments absent, small, retroverted uterus, endometriosis | Abnormal | Case Report | Level 5 |
| Mutchinick et al. 2005^56^ | 18 | amenorrhea | bilateral | ultrasound | rudimentary right tube, unicornuate uterus, absent vagina | Normal | Case Report | Level 5 |
| Mylonas et al. 2003^57^ | 28 | abdominal/ pelvic pain | right | CT scan | right tube absent, mild endometriosis | Not specified | Case Series | Level 4 |
|  | 21 | abdominal/ pelvic pain | right | - | right tube absent, rudimentary uterus | Normal |  |  |
|  | 21 | abdominal/ pelvic pain | right | - | endometriosis | Not specified |  |  |
| Okafor et al. 2013^58^ | 36 | evaluation of mass | left | ultrasound, HSG or chromotubation | left tube and broad ligament absent | Abnormal | Case Report | Level 5 |
| Ormonde et al. 2019^59^ | 31 | abdominal/ pelvic pain | left | ultrasound, CT scan | left tube absent | Normal | Case Report | Level 5 |
| Osmanagaoglu et al. 2010^60^ | 17 | amenorrhea | right | ultrasound, MRI | left tube and round ligament absent, fibroids | Abnormal | Case Report | Level 5 |
| Pabuccu et al. 2011^61^ | 21 | abdominal/ pelvic pain | left | ultrasound | left tube absent | Normal | Case Report | Level 5 |
| Peer et al. 1981^62^ | 27 | ectopic, endometrial hyperplasia | bilateral | ultrasound | no tubes bilaterally, unicornuate uterus | Normal | Case Report | Level 5 |
| Plevraki et al. 2004^63^ | Not Specified | infertility/  subfertility | left | ultrasound, HSG or chromotubation | left tube absent | Normal | Case Report | Level 5 |
|  | Not Specified | amenorrhea | bilateral | - | hypoplastic tubes, hypoplastic/rudimentary uterus | Normal |  |  |
| Rapisarda et al. 2009^64^ | 30 | amenorrhea | left | ultrasound, MRI | left tube absent, hypoplastic/rudimentary uterus | Not specified | Case Report | Level 5 |
| Rastogi et al. 2016^65^ | 25 | amenorrhea | left | ultrasound, MRI | absent tube and round ligament | Not specified | Case Report | Level 5 |
| Ruderman et al. 1962^66^ | 18 | abdominal/ pelvic pain | left | ultrasound | left tube, broad, and round ligament absent | Abnormal | Case Report | Level 5 |
| Siddigui et al. 2016^67^ | 26 | infertility/  subfertility | right | ultrasound, MRI | right tube absent | Normal | Case Series | Level 4 |
|  | 29 | abdominal/ pelvic pain | right | X-ray | right tube absent, unicornuate uterus | Normal |  |  |
|  | 28 | infertility/  subfertility | right | ultrasound, HSG or chromotubation | right tube absent | Normal |  |  |
| Silva et al. 1995^68^ | 21 | infertility/  subfertility | right | ultrasound, HSG or chromotubation | right tube absent | Not specified | Case Report | Level 5 |
| Simpson 1990^69^ | 25 | infertility/  subfertility | left | ultrasound, HSG or chromotubation | left tube blindly ending | Normal | Case Report | Level 5 |
| Sinha 1983^70^ | 17 | evaluation of mass | right | ultrasound |  | Normal | Case Report | Level 5 |
| Sirisena 1978^71^ | 19 | infertility/  subfertility | left | HSG or chromotubation |  | Normal | Case Report | Level 5 |
| Sivanesaratnam 1986^72^ | 42 | abdominal/ pelvic pain | left | - | tube absent | Normal | Case Report | Level 5 |
|  | 23 | abdominal/ pelvic pain | right | - | tube absent | Normal |  |  |
| Stanojevic et al. 2000^73^ | 16 days | sterilization | right | - | Right horn absent | Abnormal | Case Report | Level 5 |
| Suh et al. 2008^74^ | 24 | infertility/  subfertility | left | - | No UO ligament | Normal | Case Report | Level 5 |
| Sukhadiya et al. 2014^75^ | 23 | autopsy | right | ultrasound | right tube absent | Normal | Case Report | Level 5 |
| Sunku et al. 2015^76^ | 16 | infertility/  subfertility | right | ultrasound, MRI, HSG or chromotubation | tube absent, septate uterus | Not specified | Case Report | Level 5 |
| Surana et al. 1978^77^ | 1 day | infertility/  subfertility | left | ultrasound, HSG or chromotubation |  | Not specified | Case Report | Level 5 |
| Tanaka et al. 2013^78^ | 32 | amenorrhea | left | MRI | left tube absent | Normal | Case Report | Level 5 |
| Tarry et al. 1986^79^ | 7 | autopsy | right | - | right tube absent | Abnormal | Case Series | Level 4 |
|  | 17 | malignancy | right | MRI | right tube absent, arcuate uterus | Abnormal |  |  |
| Taskin et al. 2013^80^ | 32 | UTI | left | - | absent tube, uterus, and vagina | Normal | Case Report | Level 5 |
|  | 19 | amenorrhea | right | - | absent tube, rudimentary vagina | Normal |  |  |
| Topcu et al. 2013^81^ | 30 | infertility/  subfertility | right | ultrasound, CT scan, HSG or chromotubation | tube absent | Not specified | Case Report | Level 5 |
| Turner et al. 2012^82^ | 19 | abdominal/ pelvic pain | right | ultrasound |  | Not specified | Case Report | Level 5 |
| Tzitzimikas et al. 2013^83^ | 19 | foreign body | left | ultrasound,  X-ray | left tube absent, unicornuate uterus | Normal | Case Report | Level 5 |
| Vaiarelli et al. 2012^84^ | 31 | abdominal/ pelvic pain | right | - | right tube absent | Normal | Case Report | Level 5 |
| Varino et al. 1941^85^ | 27 | abdominal/ pelvic pain | left | CT scan | left tube, broad and round ligaments absent | Abnormal | Case Report | Level 5 |
| Yazawa et al. 2019^86^ | 64 | infertility/  subfertility | left | ultrasound, MRI, HSG or chromotubation | right tube absent | Not specified | Case Report | Level 5 |
| Yerebasmaz et al. 2016^87^ | 23 | abdominal/ pelvic pain | right | - | right tube absent, right ovarian mass | Normal | Case Series | Level 4 |
|  | 25 | evaluation of mass | left | CT scan, MRI,  X-ray | right tube absent | Normal |  |  |
|  | 36 | abdominal/ pelvic pain | right | ultrasound | Left adnexa absent | Normal |  |  |
| Zaitoon et al. 1982^88^ | 21 | AUB | left | ultrasound | sagital sulcus on uterine fundus | Abnormal | Case Report | Level 5 |
| Zampieri et al. 2009^89^ | Not specified | infertility/  subfertility | left | ultrasound, HSG or chromotubation | left tube absent, unicornuate uterus | Not specified | Case Report | Level 5 |
| Case included in this paper | 15 | sterilization | left | - | left tube, UO and IP ligament absent | Normal | - | - |
| Case included in this paper | 30 | evaluation of mass | left | ultrasound | right partial tube and IP | Abnormal | - | - |

1. Abargel A, Pansky M, Neeman O, Bukovsky I. Torsion of single normal adnexa in a premenarchal girl. J Am Assoc Gynecol Laparosc. 2000;7(3):421-2.

2. Alexander HD. True unicornuate uterus and total absence of left broad ligament, round ligament, salpinx, ovary, kidney and ureter. Canadian Medical Association Journal. 1947;56(5):539.

3. Ali V, Lynn S, Schmidt W. Unilateral absence of distal tube and ovary with migratory calcified intraperitoneal mass. International Journal of Gynecology and Obstetrics. 1979;17(4):328-31.

4. Alrabeeah A, Galliani CA, Giacomantonio M, Heifetz SA, Lau H. Neonatal ovarian torsion: Report of three cases and review of the literature. Pediatric Pathology. 1988;8(2):143-9.

5. Alvir I, Puljiz M, Tomica D, Danolic D, Mamic I, Toth T. An incidental finding of unicornuate uterus with unilateral ovarian agenesis during laparoscopy in patient who gave birth to eleven children: A case report. Collegium Antropologicum. 2013;37(1):289-91.

6. Aslam A, Wong C, Haworth JM, Noblett HR. Autoamputation of ovarian cyst in an infant. J Pediatr Surg. 1995;30(11):1609-10.

7. Awad C, Damavandy T. Incidental finding of autoamputated, free-floating ovary by laparoscopy: A case report. Journal of Reproductive Medicine. 2018;63(2):168-70.

8. Barsky M, Beaulieu AM, Sites CK. Congenital ovarian-fallopian tube agenesis predisposes to premature surgical menopause: A report of two cases. J Androl Gynaecol. 2015;3(1):3.

9. Bates GW, Abide JK. Bilateral autoamputation of the fallopian tubes. Fertility and Sterility. 1982;38(2):253-4.

10. Bay UO, Gun BD, Ungan B, Dogan S, Barut A, Arikan II. Unilateral ovarian agenesis and clear cell type epithelioid leiomyoma of uterus mimicking ovarian malignancy. Journal of the Pakistan Medical Association. 2014;64(1).

11. Ben-Nun I, Fejgin M, Gruber A, Ben-Aderet N. Transperitoneal ovum migration in women with unilateral congenital ovarian absence. Acta Obstetricia et Gynecologica Scandinavica. 1988;67(7):665-7.

12. Biljic-Erski IR, Vasiljevic M, Rakic S, Mihajlovic S. Unilateral agenesis of the right ovary and Fallopian tube in an infertile patient with a normal uterus. Vojnosanitetski Pregled. 2019;76(6):641-4.

13. Blumberg K, Wood BP. Radiological case of the month. Amputated calcified ovary. Am J Dis Child. 1993 May;147(5):585-6.

14. Bousfiha N, Errarhay S, Saadi H, Ouldim K, Bouchikhi C, Banani A. Gonadal dysgenesis 46, XX associated with Mayer-Rokitansky-Kuster-Hauser syndrome: one case report. Obstetrics and Gynecology International. 2010;2010.

15. Bradley B, Gleicher N. Grand multiparity associated with unilateral renal, ovarian and Mullerian agenesis. Mount Sinai Journal of Medicine. 1980;47(4):418-22.

16. Bugmann P, Hanquinet S, Brundler MA, Birraux J, Genin B, Le Coultre C. Intestinal obstruction caused by an ectopic fallopian tube in a child: Case report and literature review. Journal of Pediatric Surgery. 2001;36(3):508-10.

17. Burge ES. Absence of left ovary and portion of left fallopian tube in 19-year-old student; case report. Quarterly Bulletin. 1958;32(1):4-5.

18. Castellani C, Petnehazy T, Gurtl-Lackner B, Saxena AK. A Rare Cause for a Neonatal Cystic Abdominal Mass. Journal of Minimally Invasive Gynecology. 2013;20(5):714-6.

19. Chan CLK, Leeton JF. A case report of bilateral absence of fallopian tubes and ovaries. Asia‐Oceania Journal of Obstetrics and Gynaecology. 1987;13(3):269-71.

20. Chen B, Yang C, Sahebally Z, Jin H. Unilateral ovarian and fallopian tube agenesis in an infertile patient with a normal uterus. Experimental and Therapeutic Medicine. 2014;8(3):831-5.

21. Choudhary V, Sharma P. A Case Series Depicting Agenesis Of Uterine Adenexa At Different Age Groups.

22. Cucinella G, Billone V, Misseri G, Rotolo S, Triolo MM, Pitruzzella I, et al. Interstitial pregnancy in a patient with ipsilateral adnexal absence: Case report and literature review. Giornale Italiano di Ostetricia e Ginecologia. 2013;35(6):733-9.

23. Currarino G, Rutledge JC. Ovarian torsion and amputation resulting in partially calcified, pedunculated cystic mass. Pediatr Radiol. 1989;19(6-7):395-9.

24. Dannreuther WT. Dextroversion of the uterus with congenital absence lift fallopian tube, ovary, broad ligament, round ligament, kidney and ureter. American Journal of Obstetrics and Gynecology. 1923;6:51-3.

25. Dare FO, Makinde OO, Makinde ON, Odutayo R. Congenital absence of an ovary in a Nigerian woman. International Journal of Gynecology and Obstetrics. 1989;29(4):377-8.

26. De AB, V.; Gupta, N. Type-2 Mayer-Rokitansky-Kuster-Hauser Syndrome with Periureteric Fibrosis: A Rare Association. Pan Asian Journal of Obstetrics & Gynecology. 2019;2(1):49-52.

27. Dede M, Gezginc K, Ulubay M, Alanbay I, Yenen M. A rare case of rudimentary uterus with absence of both ovaries and 46,XX normal karyotype without mosaicism. Taiwanese Journal of Obstetrics and Gynecology. 2008;47(1):84-6.

28. Demir B, Guven S, Guvendag Guven ES, Gunalp GS. An incidental finding of unicornuate uterus with unilateral ovarian agenesis during cesarean delivery. Archives of Gynecology and Obstetrics. 2007;276(1):91-3.

29. Dueck A, Poenaru D, Jamieson MA, Kamal IK. Unilateral ovarian agenesis and fallopian tube maldescent. Pediatric Surgery International. 2001;17(2-3):228-9.

30. Durous V, Milot L, Buy JN, Deval B, Rousset P. Auto-amputated adnexa in a young woman: Multimodal imaging to rule in a pelvic rolling stone. Journal of Gynecology Obstetrics and Human Reproduction. 2019;48(6):423-6.

31. Eda M, Kaidoh T, Takanashi Y, Inoué T. A stone-like ovarian dermoid cyst in the Douglas' Pouch of an elderly woman. Pathol Int. 2012;62(11):771-3.

32. Eustace DLS. Congenital absence of fallopian tube and ovary. European Journal of Obstetrics Gynecology and Reproductive Biology. 1992;46(2-3):157-9.

33. Fletcher RM, Boal DK, Karl SR, Gross GW. Ovarian torsion: an unusual cause of bilateral pelvic calcifications. Pediatr Radiol. 1988;18(2):172-3.

34. Focseneanu MA, Omurtag K, Ratts VS, Merritt DE. The Auto-Amputated Adnexa: A Review of Findings in a Pediatric Population. Journal of Pediatric and Adolescent Gynecology. 2013;26(6):305-13.

35. Galli PAP, D. Ipsilateral Fallopian Tube and Ovary Agenesis or Absence? Case Report and Review of the Literature. Journal of Gynecology and Womens Health. 2017 12/18;7.

36. Guthrie D, Wilson LB. X. Congenital Unilateral Absence of the Urogenital System. Annals of surgery. 1909;50(5):907.

37. Gutierrez R. Surgical aspects of renal agenesis: with special reference to hypoplastic kidney, renal aplasia and congenital absence of one kidney. Archives of Surgery. 1933;27(4):686-735.

38. Haydardedeoglu B, Simsek E, Kilicdag EB, Tarim E, Aslan E, Bagis T. A case of unicornuate uterus with ipsilateral ovarian and renal agenesis. Fertil Steril. 2006 Mar;85(3):750.e1-.e4.

39. Kennedy LA, Pinckney LE, Currarino G, Votteler TP. Amputated calcified ovaries in children. Radiology. 1981;141(1):83-6.

40. Kent BK. Ectopic pregnancy in a congenitally defective tube with absence of the ipsolateral ovary. American Journal of Obstetrics and Gynecology. 1956;72(5):1150-1.

41. Koh KS. Letter: Absence of uterine adnexa. Can Med Assoc J. 1974;111(12):1304.

42. Kriplani A, Takkar D, Karak AK, Ammini AC. Unexplained absence of both fallopian tubes with ovary in the omentum. Archives of Gynecology and Obstetrics. 1995;256(2):111-3.

43. Kumar A, Mishra S, Dogra PN. Management of an unusual case of atypical Mayer-Rokitansky-Kuster-Hauser syndrome, with unilateral gonadal agenesis, solitary ectopic pelvic kidney, and pelviureteric junction obstruction. International Urogynecology Journal. 2007;18(7):823-5.

44. Kurcz JA, Sharp MS. Congenital absence of one ovary associated with contralateral tubal pregnancy. American Journal of Obstetrics and Gynecology. 1948;55(6):1065-7.

45. Kusaka M, Mikuni M. Ectopic ovary: A case of autoamputated ovary with mature cystic teratoma into the cul-de-sac. Journal of Obstetrics and Gynaecology Research. 2007;33(3):368-70.

46. Lashgari M. Letter: Absence of ovary. Obstetrics and Gynecology. 1975;46(1):115-6.

47. Lee KH, Song MJ, Jung IC, Lee YS, Park EK. Autoamputation of an ovarian mature cystic teratoma: a case report and a review of the literature. World Journal of Surgical Oncology. 2016;14:7.

48. Liu Q, Liu HQ, Jiang YY, Sun XB. Is unilateral uterine adnexa absence a congenital developmental abnormality or posteriority? Summary of 39 cases and literature review. Archives of Gynecology and Obstetrics. 2013;288(3):555-61.

49. Loo ZX, Long CY, Jeng CJ. Extragonadal mature cystic teratoma at anterior uterine wall coexisting with a pararectal corpus luteum cyst and agenesis of left fallopian tube and left ovary. Taiwanese Journal of Obstetrics and Gynecology. 2020;59(1):154-6.

50. Luvero D, Plotti F, Capriglione S, Miranda A, Lopez S, Scaletta G, et al. Undescended or absent ovary without uterine anomalies: systematic review and a single-center experience. Minerva Ginecologica. 2016;11:11.

51. Mecklenburg RS, Krueger PM. Extensive Genitourinary Anomalies Associated with Klippel-Feil Syndrome. American Journal of Diseases of Children. 1974;128(1):92-3.

52. Metoki R, Orikasa S, Ohta S, Kanetoh H. A case of bladder agenesis. Journal of Urology. 1986;136(3):662-4.

53. Mishell DR. Unilateral absence of the fallopian tube and ovary. American Journal of Obstetrics and Gynecology. 1938;36(4):705-6.

54. Morao S, Chaves F, Virella D, Alves F, Alves R, Pascoal J. MURCS association and anorectal malformation: Case report of a female newborn. Journal of Pediatric Surgery Case Reports. 2017;18:19-23.

55. Muppala H, Sengupta S, Martin JE. Unilateral absence of tube and ovary with renal agenesis and associated pyloric stenosis: Communication. European Journal of Obstetrics and Gynecology and Reproductive Biology. 2008;137(1):123.

56. Mutchinick OM, Morales JJ, Zenteno JC, Del Castillo CF. A rare case of gonadal agenesis with paramesonephric derivatives in a patient with a normal female karyotype. Fertility and Sterility. 2005;83(1):201-4.

57. Mylonas I, Hansch S, Markmann S, Bolz M, Friese K. Unilateral ovarian agenesis: Report of three cases and review of the literature. Archives of Gynecology and Obstetrics. 2003;268(1):57-60.

58. Okafor CO, Okafor CI, Oguaka VN, Obionwu IC. Unilateral absence of the Fallopian tube and ovary with ipsilateral renal agenesis. Nigerian Journal of Surgical Sciences. 2013;23(1):21.

59. Ormonde M, Raposo MI, Cardoso M, Pereira A, Demendonca R. Unilateral ovarian and fallopian tube agenesis. Gazzetta Medica Italiana Archivio per le Scienze Mediche. 2019;178(4):234-7.

60. Osmanağaoğlu M, Güven S, Aran T, Özgür M, Vurallı B, İlhan CG, et al. Uterus Unicornis and Unilateral Ovarian and Renal Agenesis in A Case with Primary Amenorrhoea. Gynecology Obstetrics & Reproductive Medicine. 2010;16(2):127-9.

61. Pabuccu E, Kahraman K, Taskn S, Atabekoglu C. Unilateral absence of fallopian tube and ovary in an infertile patient. Fertility and Sterility. 2011;96(1):e55-e7.

62. Peer E, Kerner H, Peretz BA, Makler A, Paldi E. Bilateral adnexal agenesis with an ectopic ovary - Case report and review of the literature. European Journal of Obstetrics Gynecology and Reproductive Biology. 1981;12(1):37-42.

63. Plevraki E, Kita M, Goulis DG, Hatzisevastou-Loukidou H, Lambropoulos AF, Avramides A. Bilateral ovarian agenesis and the presence of the testis-specific protein 1-Y-linked gene: Two new features of Mayer-Rokitansky-Kuster-Hauser syndrome. Fertility and Sterility. 2004;81(3):689-92.

64. Rapisarda G, Pappalardo EM, Arancio A, La Greca M. Unilateral ovarian and fallopian tube agenesis. Archives of Gynecology and Obstetrics. 2009;280(5):849-50.

65. Rastogi R, Gupta Y, Gupta B, Sinha P, Chaudhary M, Parashar S, et al. Unilateral Agenesis of Adnexa–A Rare Clinico-Radiological Condition. J Med Diagn Meth. 2016;5(228):2.

66. Ruderman RL, Mayer JM. Unilateral renal agenesis with unicornuate uterus. Canadian Medical Association Journal. 1962;87(5):235.

67. Siddiqui M, Ghafoor N, Naznine F, Siddiqua CA, Chowdhury T. Unilateral absence of ovary and fallopian tube: 3 rare case reports. Bangladesh Journal of Obstetrics and Gynecology. 2016;31(2):97-100.

68. Silva PD, Glasser KE, Virata RL. Spontaneously acquired, unilateral absence of the adnexa: A case report. Journal of Reproductive Medicine for the Obstetrician and Gynecologist. 1995;40(1):63-4.

69. Simpson CW. Concomitant Unilateral Ovarian-Fallopian Tube Abnormality. Journal of Gynecologic Surgery. 1990;6(2):115-8.

70. Sinha MR. Unexplained absence of a fallopian tube and an ovary. Journal of the Indian Medical Association. 1983;80(7-8):103-4.

71. Sirisena LAW. Unexplained absence of an ovary and uterine tube. Postgraduate Medical Journal. 1978;54(632):423-4.

72. Sivanesaratnam V. Unexplained unilateral absence of ovary and fallopian tube. European Journal of Obstetrics Gynecology and Reproductive Biology. 1986;22(1-2):103-5.

73. Stanojevic M, Stipoljev F, Koprcina B, Kurjak A. Oculo-auriculo-vertebral (Goldenhar) spectrum associated with pericentric inversion 9: Coincidental finding or etiologic factor? Journal of Craniofacial Genetics and Developmental Biology. 2000;20(3):150-4.

74. Suh BY, Kalan MJ. Septate uterus with left fallopian tube hypoplasia and ipsilateral ovarian agenesis. Journal of Assisted Reproduction and Genetics. 2008;25(11-12):567-9.

75. Sukhadiya M, Grover SV. Unexplained unilateral absence of fallopian tube and ovary: A rare occurrence. Journal of SAFOMS. 2014;2(1):46.

76. Sunku R, Duggal R, Patel FD, Rai B, Srinivasan R, Nijhawan R. Cervical embryonal rhabdomyosarcoma and ovarian Sertoli-Leydig cell tumor with congenital absence of unilateral ovary. Journal of Cancer Research and Therapeutics. 2015;11(3):654.

77. Surana RB, Fraga JR, Sinkford SM. The cerebro-oculo-facio-skeletal syndrome. Clinical Genetics. 1978;13(6):486-8.

78. Tanaka Y, Koyama S, Kobayashi M, Kubota S, Nakamura R, Isobe M, et al. Complex Mullerian malformation without any present classification: unilateral ovarian and tubal absence with an arcuate uterus. Asian journal of endoscopic surgery. 2013;6(1):55-7.

79. Tarry WF, Duckett JW, Stephens FD. The Mayer-Rokitansky syndrome: Pathogenesis, classification and management. Journal of Urology. 1986;136(3):648-52.

80. Taşkin EA, Cirik DA, Çalişkan AC, Ortaç A, Tuǧlu A. Unilateral absence of the fallopian tube and the ovary: Report of two cases and brief review of the literature. Journal of Gynecologic Surgery. 2013;29(2):72-5.

81. Topcu HO, Esercan A, Kokanali MK, Sarikaya E, Evliyaoglu O. Complete absence of fallopian tube and adjacent ovary in a fertile patient. Journal of Experimental and Clinical Medicine (Turkey). 2017;34(2):129-31.

82. Turner EJH, Thomas PRS. Absent ovary and peritoneal stone found at laparoscopy. BMJ Case Reports. 2012.

83. Tzitzimikas S, Fragkos M, Karavida A, Mettler L. Unilateral ovarian absence. Gynecological Surgery. 2013;10(1):93-5.

84. Vaiarelli A, Luk J, Patrizio P. Ectopic pregnancy after IVF in a patient with unilateral agenesis of the fallopian tube and ovary and with endometriosis: Search of the literature for these associations. Journal of Assisted Reproduction and Genetics. 2012;29(9):901-4.

85. Varino GA, Beacham WD. Left renal agenesis, true unicornuate uterus, and total absence of left broad ligament, round ligament, salpinx, and ovary. American Journal of Obstetrics and Gynecology. 1941;41:124-8.

86. Yazawa H, Takiguchi K, Kato A, Imaizumi K. An unusual presentation of ovarian fibroma originating from an autoamputated ovary. Gynecology and Minimally Invasive Therapy-Gmit. 2019;8(1):40-3.

87. Yerebasmaz N, Dilbaz B, Sengul O, Altinbas S, Cakir L. Four cases with congenital unilateral absence of ovary and fallopian tube: Review of the literature. Journal of Clinical and Analytical Medicine. 2016;7(Supplement 3):275-8.

88. Zaitoon MM, Florentin H. Crossed renal ectopia with unilateral agenesis of fallopian tube and ovary. Journal of Urology. 1982;128(1):111.

89. Zampieri N, Scirè G, Zamboni C, Ottolenghi A, Camoglio FS. Unusual Presentation of Antenatal Ovarian Torsion: Free-Floating Abdominal Cysts. Our Experience and Surgical Management. Journal of Laparoendoscopic and Advanced Surgical Techniques. 2009;19(SUPPL. 1):S149-S52.

**Supplementary Table 4: Excluded Studies Table with reasons for Exclusion**

| **First Authors Last Name** | **Year** | **Title** | **Journal** | **Reason for Exclusion** |
| --- | --- | --- | --- | --- |
| Abaci | 2007 | Case report of two siblings with familal ovarian dysgenesis | Minerva Pediatrica | Ovarian Anomaly Not Determined |
| Abdelazim | 2021 | Inevitable removal of left accessory ovary | Gynecology and Minimally Invasive Therapy | No Original Data |
| Abedalthagafi | 2009 | Primary retroperitoneal mucinous cystadenoma | Saudi Medical Journal | Ineligible Patient Population |
| Abir | 2003 | Severe ovarian dysgenesis and enlarged dysplastic kidneys in two siblings with normal karyotypes | Fertility and Sterility | Ineligible Patient Population |
| Abrego | 1975 | Mesenteric supernumerary ovary | Obstetrics and Gynecology | Ectopic Ovaries |
| Acar | 2019 | A Case Report: Neonatal Torsional Ovarian Cyst | Medical Bulletin of Sisli Etfal Hospital | Ectopic Ovaries |
| Acien | 2006 | The importance of being precise about Mullerian malformations | Fertility and Sterility | No Original Data |
| Agbor | 2016 | A rare presentation of ectopic ovary in a female adolescent and the impact of obesity: a case report | Journal of Medical Case Reports | Duplicate |
| Agbor | 2016 | A rare presentation of ectopic ovary in a female adolescent and the impact of obesity: A case report | Journal of Medical Case Reports | Ectopic Ovaries |
| Alahiri | 2020 | Late-onset hepatocyte nuclear factor 1beta-associated kidney disease | Journal of the American Society of Nephrology | Conference Abstract |
| Albeaux-Fernet | 1957 | [Ovarian agenesis] | Gaz Med Fr | Non-english language |
| Allen | 2012 | Incidence of Ovarian Maldescent in Women With Mullerian Duct Anomalies: Evaluation by MRI | American Journal of Roentgenology | Ineligible Patient Population |
| Alli | 2008 | Left retroperitoneal paraganglioma with enlarged ovarian vessels | European Journal of Radiology Extra | Ectopic Ovaries |
| AlOmari | 2011 | Inguinal uterus, fallopian tube, and ovary associated with adult Mayer-Rokitansky-Kuster-Hauser syndrome | Fertility and Sterility | Ectopic Ovaries |
| AlonsoJimenez | 2009 | Intrauterine torsion of ovarian cyst. Atypical neonatal presentation. [Spanish] | Acta Pediatrica Espanola | Ectopic Ovaries |
| Alpern | 1986 | Coexistence of gonadal dysgenesis and uterine aplasia. A case report | J Reprod Med | Ineligible Patient Population |
| Alpern | 1990 | Supernumerary ovary. A case report | Journal of Reproductive Medicine for the Obstetrician and Gynecologist | Ectopic Ovaries |
| Alsina | 2021 | Incidental Finding of Unilateral Ovarian and Fallopian Tube Agenesis During Cesarean Delivery in Patient With Recurrent Pregnancy Loss | Cureus | Ectopic Ovaries |
| Altay | 2010 | Laparoscopic management of primary abdominal pregnancy: A case report | Journal of the Turkish German Gynecology Association | Ectopic Ovaries |
| Altinay-Kirli | 2017 | A rare cause of abdominal pain: Ectopic ovary and intestinal malrotation | The Turkish journal of pediatrics | Ectopic Ovaries |
| Alturkistani | 2016 | Mayer-rokitansky-kuster-hauser syndrome; spectrum of presentation and the utility of daignosis using us and MRI | Pediatric Radiology | Conference Abstract |
| AlvarezGil | 2015 | A case of clear cell carcinoma in an accessory ovary. [Spanish] | Progresos de Obstetricia y Ginecologia | Ectopic Ovaries |
| Amarin | 1988 | Inguinal ovary and fallopian tube--an unusual hernia | Int J Gynaecol Obstet | Ectopic Ovaries |
| Andrade | 2001 | Sclerosing stromal tumor in an accessory ovary | Gynecologic Oncology | Ectopic Ovaries |
| Anonymous | 2012 | NASPAG 26th Annual Clinical Research Meeting | Journal of Pediatric and Adolescent Gynecology. | Ectopic Ovaries |
| Anonymous | 1952 | MALFORMATIONAL syndrome suggesting ovarian agenesis in preadolescence | Journal of Pediatrics | Ineligible Patient Population |
| Arakelyan | 2021 | A rare concurrence of congenital ovarian and uterine malformations. [Russian] | Akusherstvo i Ginekologiya | Ectopic Ovaries |
| Archangelo | 2014 | Videolaparoscopic approach of the dermoid cyst in the omentum: A case report | Journal of Minimally Invasive Gynecology | Ectopic Ovaries |
| Atakul | 2009 | Klippel-Feil anomaly associated, Rokitansky-Kudie;stner syndrome, pelvic kidneys and bilateral gonadal dysgenesis: A case report | International Journal of Gynecology and Obstetrics | Ineligible Patient Population |
| Azam | 1999 | Retroperitoneal mucinous cystadenoma. A case report | Progres en Urologie | Ectopic Ovaries |
| Ba'Aqeel | 1988 | Unilateral streaked ovary (Slotnick-Goldfarb) syndrome: A case report and review of the literature | Annals of Saudi Medicine | Ineligible Patient Population |
| Badawy | 1995 | Supernumerary ovary with an endometrioma and osseous metaplasia: A case report | American Journal of Obstetrics and Gynecology | Ectopic Ovaries |
| Bae | 2013 | Cancer of the supernumerary ovary in mayer-rokitansty-kuster-hauser syndrome: A case report | Oncology Letters | Ectopic Ovaries |
| Baek | 2019 | Teratoma of the Omentum - An Incidental Finding | Gynakologische Endokrinologie | Ectopic Ovaries |
| Bagga | 2018 | Functioning left uterine horn with cervico-vaginal atresia and ovarian maldescent - an unclassified Mullerian anomaly treated with horn-vaginal anastomosis | Journal of Obstetrics & Gynaecology | Ectopic Ovaries |
| Baje | 2020 | Cervical dysgenesis with unicornuate uterus: A case report | Journal of Obstetrics and Gynaecology Research | Ovarian Anomaly Not Determined |
| Balak | 1977 | Agenesis ovarii 1. dx. cum atresia tubae 1. dx. [Czech] | Ceskoslovenska Gynekologie | Non-english language |
| BanuelosMartinez | 2016 | Unilateral ovarian agenesis and malformations in a pregnant woman with hyperimmunoglobulin E syndrome | ClÃ­nica e InvestigaciÃ³n en GinecologÃ­a y Obstetricia | Non-english language |
| Barakat | 2002 | Association of unilateral renal agenesis and genital anomalies | Case Rep Clin Pract Rev | Ineligible Patient Population |
| Barik | 1991 | Adenocarcinoma of the supernumerary ovary | International Journal of Gynecology and Obstetrics | Ectopic Ovaries |
| Barnts | 2022 | Tubal pregnancy in a case of unicornuate uterus with contralateral streak ovary and renal agenesis: A case report | Case Reports in Women's Health | Ineligible Patient Population |
| Bawahab | 2017 | Isolated left descended inguinal ovary with ipsilateral ectopic pelvic kidney: A case report and review of literature | Clinical and Experimental Obstetrics and Gynecology | Ectopic Ovaries |
| Bayramov | 2009 | Ectopic ovary autotransplanted over rectosigmoid colon: a case report | Fertility and Sterility | Ectopic Ovaries |
| Bazi | 2007 | Inguinal ovary after controlled ovarian hyperstimulation-infrequent, infrequently recognized, or observer variation? | Fertility and Sterility | No Original Data |
| Bazi | 2006 | Inguinal ovaries associated with mullerian agenesis: case report and review | Fertility and Sterility | Ectopic Ovaries |
| Behrendt | 2016 | The results of the treatment of ovarian cysts in neonates 5-year observations | Pediatria Polska | Ectopic Ovaries |
| Bellver-Pradas | 2001 | Silver-Russell syndrome associated to Mayer-Rokitansky-Kuster-Hauser syndrome, diabetes and hirsutism | Archives of Gynecology and Obstetrics | Ectopic Ovaries |
| Benbara | 2011 | Accessory ovary in the utero-ovarian ligament: An incidental finding | Archives of Gynecology and Obstetrics | Ectopic Ovaries |
| Berrada | 2019 | Bilateral tubal agenesis: A case report | International Journal of Innovation and Applied Studies | Ineligible Patient Population |
| Bertrand | 1956 | Ovarian agenia | Comptes Rendus de la Societe Francaise de Gynecologie | Non-english language |
| Besser | 1992 | Cystic teratoma in a supernumerary ovary of the greater omentum: A case report | Journal of Reproductive Medicine for the Obstetrician and Gynecologist | Ectopic Ovaries |
| Bhandari | 2020 | A case of dorsal agenesis of pancreas associated with unilateral renal agenesis, unicornuate uterus, and ovarian ectopia: A brief review and learning points | Indian Journal of Radiology and Imaging | Ineligible Patient Population |
| Bidus | 2004 | Mayer-Rokitansky-Kuster-Hauser syndrome presenting as an inguinal mass and hernia in the female patient | Journal of Pelvic Medicine and Surgery | Ectopic Ovaries |
| Bizzarri | 2020 | Unusual Presentation of a Denys-Drash Syndrome Girl with Undisclosed Assumption of Biotin | Journal of clinical research in pediatric endocrinology. | Ectopic Ovaries |
| Bjorsum-Meyer | 2016 | Vertebral defect, anal atresia, cardiac defect, tracheoesophageal fistula/esophageal atresia, renal defect, and limb defect association with Mayer-Rokitansky-Kuster-Hauser syndrome in co-occurrence: two case reports and a review of the literature | Journal of Medical Case Reports | Ineligible Patient Population |
| Blais | 2019 | The kidney that wasn't | Journal of Urology | Ovarian Anomaly Not Determined |
| Bleach | 2020 | A Case of Mayer-Rokitansky-Kuster-Hauser Syndrome Diagnosed in Infancy after Evaluation of Palpable Gonads | Journal of Pediatric and Adolescent Gynecology | Ectopic Ovaries |
| Bleha | 1953 | [Shereshevskii's syndrome; ovarian agenesis, Turner-Albright's syndrome] | Casopis Lekaru Ceskych | Ineligible Patient Population |
| Bolomini | 2020 | Twist and re-twist of the ovary in a young woman with ribbon-like contralateral ovary and absence of contralateral tube | Ultrasound in Obstetrics & Gynecology | Ineligible Patient Population |
| Bolomini | 2021 | Repeat twisting of ovary in young woman with ribbon-like contralateral ovary and absence of contralateral Fallopian tube | Ultrasound in Obstetrics & Gynecology | Ineligible Patient Population |
| Bonilla-Ruvalcaba | 2021 | Mutations of the hepatocyte nuclear factor gene 1β (HNF1β) as a cause of kidney, liver and diabetes damage | Revista Mexicana de Pediatria | Non-english language |
| BorosDohan | 2015 | Extragonadal dermoid cyst of the great omentum: An incidental finding | Virchows Archiv | Ectopic Ovaries |
| Borruto | 1999 | Adnexal malformations: Anatomical and clinical considerations. [Italian] | Rivista di Ostetricia e Ginecologia | Ectopic Ovaries |
| Bradshaw | 1986 | Ovarian and tubal inguinal hernia | Obstetrics and Gynecology | Ectopic Ovaries |
| Brady | 1925 | An Adenomyoma of the vesico-vaginal septum and a supernumerary ovary | Bulletin of the Johns Hopkins Hospital | Ectopic Ovaries |
| Breborowicz | 2012 | Unilateral ovarian hypoplasia a report of two cases |  | Ineligible Patient Population |
| Brucker | 2020 | Living-Donor Uterus Transplantation: Pre-, Intra-, and Postoperative Parameters Relevant to Surgical Success, Pregnancy, and Obstetrics with Live Births | Journal of Clinical Medicine | Ineligible Patient Population |
| Burnett | 1961 | Supernumerary ovary. A case report | American Journal of Obstetrics & Gynecology | Ectopic Ovaries |
| Burns | 1968 | Followup on previously reported congenital absence of gonads | Transactions of the American Association of Genito-Urinary Surgeons | Ineligible Patient Population |
| CarullaRoig | 2015 | Delay in the diagnosis of Rokitansky syndrome due to an early onset anorexia nervosa. Case report and review of the literature | European Child and Adolescent Psychiatry | Conference Abstract |
| Ceci | 1967 | [Unilateral Wolff-Mullerian agenesis associated with homolateral gonadal agenesis] | Atti della Accademia Dei Fisiocritici in Siena - Sezione Medico-Fisica | Non-english language |
| Cerekja | 2011 | Unicornuate uteri associated with contralateral renal agenesis and ovarian anomalies | Journal of the Turkish German Gynecology Association | Ineligible Patient Population |
| Chen | 2021 | 86. Unilateral Ovarian Agenesis: A Systematic Review of the Literature and Report of Two Cases | Journal of Pediatric and Adolescent Gynecology | Conference Abstract |
| Chen | 2008 | SPONTANEOUS RUPTURE OF OMENTAL TERATOMA MIMICKING A RUPTURED OVARIAN TERATOMA | Taiwanese Journal of Obstetrics & Gynecology | Ineligible Patient Population |
| Cheng | 2019 | Ovarian insufficiency: The hidden uterus | Hormone Research in Paediatrics | Ineligible Patient Population |
| Cheng | 2021 | Menarche in primary ovarian insufficiency after a month of hormone replacement therapy: a case report | Journal of Medical Case Reports | Ineligible Patient Population |
| Chernyshova | 2019 | Combination of cervical cancer and unilateral complete aplasia of the adnexa: a case report | Byulleten Sibirskoy Meditsiny | Ineligible Patient Population |
| Cheung | 2010 | Accessory ovary | Journal of Obstetrics & Gynaecology Canada: JOGC | Ectopic Ovaries |
| Chirico | 1959 | [Chromosomal sex and ovarian agenesis. Contribution and pathogenetic considerations] | Minerva Medica | Non-english language |
| Chitrambalam | 2020 | Torsion Adnexal Mass in a Patient with Mullerian Agenesis | Journal of Evolution of Medical and Dental Sciences | Ineligible Patient Population |
| Ciccone | 2018 | A 46,XX female with WT1 mutation, congenital nephrotic syndrome and a complex disorder of sex development | Hormone Research in Paediatrics | Ectopic Ovaries |
| Cloke | 2017 | An unusual case of primary amenorrhoea-concurrent ovarian dysgenesis and Mayer-Rokitansky-Kuster-Hauser syndrome | Bjog-an International Journal of Obstetrics and Gynaecology | Conference Abstract |
| Cohen | 2001 | A giant ectopic ovary | Journal of Laparoendoscopic and Advanced Surgical Techniques - Part A | Ectopic Ovaries |
| Colombani | 2007 | Fourth case of uterine aplasia, ovarian dysgenesis, amenorrhea and impuberism: a variant of Mayer-Rokitansky-Kuster-Hauser syndrome | Acta Paediatrica | Ineligible Patient Population |
| Cottrill | 2007 | Primary retroperitoneal mucinous borderline tumor: A case report | Gynecologic Oncology | Ectopic Ovaries |
| Coursimault | 2019 | Uterine and ovarian agenesis in a girl with LIG4 syndrome | European Journal of Human Genetics | Conference Abstract |
| Cripps | 1838 | PERFORATION OF THE STOMACH PERITONITIS. ABSENCE OF THE OVARIES | The Lancet | Ectopic Ovaries |
| Cruikshank | 1982 | Supernumerary ovaries: Update and review | Obstetrics and Gynecology | Ectopic Ovaries |
| Cruikshank | 1991 | Supernumerary ovary: Embryology | International Journal of Gynecology and Obstetrics | Ectopic Ovaries |
| Cutler | 1953 | Ovarian agenesis (congenitally aplastic ovaries) in a 3-year-old female | Journal of Pediatrics | Ineligible Patient Population |
| Dabirashrafi | 1994 | Ovarian malposition in women with uterine anomalies | Obstetrics & Gynecology | Ectopic Ovaries |
| Dabkowska-Huc | 2013 | MURCS Association with Partial Duplication of the Distal Long Chromosome 5 and Unilateral Ovarian Agenesis | Case Reports in Genetics | Ineligible Patient Population |
| Daw | 1986 | Absence of an ovary and uterine tube - Further evidence for the 'torsion' theory | Journal of Obstetrics and Gynaecology | Ineligible Patient Population |
| DeCaro | 1997 | Renal agenesis associated with homolateral ovarian dysplasia. A case report. [Italian] | La Pediatria medica e chirurgica : Medical and surgical pediatrics | Ectopic Ovaries |
| Decker | 1999 | Laparoscopic diagnosis and management of ovarian torsion in the newborn | JSLS : Journal of the Society of Laparoendoscopic Surgeons / Society of Laparoendoscopic Surgeons | Ectopic Ovaries |
| Delecour | 1966 | [Ectopic lumbar ovary and complications] | Bulletin de la Federation des Societes de Gynecologie et d Obstetrique de Langue Francaise | Ectopic Ovaries |
| DeMarchena | 1952 | [Twisted ectopic ovary on the left inguinal canal in a four month old girl] | Archivos de Medicina Infantil | Ectopic Ovaries |
| Demirel | 2012 | Inguinal ovary as a rare diagnostic sign of Mayer-Rokitansky-Kuster- Hauser syndrome | Journal of Pediatric Endocrinology and Metabolism | Ectopic Ovaries |
| Derevianko | 1988 | A giant cyst of ectopic ovarian tissue. [Russian] | Klinicheskaia khirurgiia | Ectopic Ovaries |
| Desorgher | 1965 | [Complications of Ectopic Ovary. Apropos of 2 Cases] | Journal des Sciences Medicales de Lille | Ectopic Ovaries |
| Desteli | 2013 | Undescended ovary presented with paraovarian cyst | Turk Jinekoloji ve Obstetrik Dernegi Dergisi | Ectopic Ovaries |
| Detrehazy | 1955 | [Inguinal ectopic ovary] | Orvosi Hetilap | Ectopic Ovaries |
| DeVink | 1949 | Agenesis of the ovary | Nederlandsch Tijdschrift voor Verloskunde en Gynaecologie | Non-english language |
| DeVink | 1949 | Agenesis of the ovary | Nederlandsch Tijdschrift voor Verloskunde en Gynaecologie | Non-english language |
| DeWet | 1958 | Unicornuate uterus with ectopic teratomatous ovary and ectopic kidney | South African Medical Journal. Suid-Afrikaanse Tydskrif Vir Geneeskunde | Ectopic Ovaries |
| Dgheem | 2014 | Ectopic ovary: One new location | Surgical Techniques Development | Ectopic Ovaries |
| Dikaiakou | 2021 | Two sisters with primary hypergonadotrophic hypogonadism, pubertal progress and deletion of 61.5MB of Xq21.33q28 region | Hormone Research in Paediatrics | Conference abstract |
| Dillon | 1981 | A case of accessory ovary | Obstetrics and Gynecology | Ectopic Ovaries |
| Dmowski | 1972 | Unilateral ovarian dysgenesis with prenatal virilization | Obstetrics and Gynecology | Ineligible Patient Population |
| Dodion | 1969 | [Marfan's syndrome with mitral insufficiency, mental retardation and ovarian agenesis] | Acta Paediatrica Belgica | Non-english language |
| Dragusin | 2014 | Importance of Laparoscopic Assessment of the Uterine Adnexa in a Mayer-Rokitansky-Kuster-Hauser Syndrome Type II Case | Current Health Sciences Journal | Ectopic Ovaries |
| Dreuning | 2020 | Inguinal hernia in girls: A retrospective analysis of over 1000 patients | J Pediatr Surg | Ineligible Patient Population |
| Drummond | 1939 | Unilateral renal agenesis with associated genital anomalies | The Journal of Urology | Ovarian Anomaly Not Determined |
| Dubois | 1953 | On the excretion of oestrogens in the urine of patients with ovarial agenesia | Acta Physiologica et Pharmacologica Neerlandica | Ineligible Patient Population |
| Edwards | 2018 | The Management of an Ectopic Ovary in an Adolescent Patient with Gastroschisis | Journal of Pediatric and Adolescent Gynecology | Ectopic Ovaries |
| El-Gohary | 2015 | Supernumerary ovary presenting as a paraduodenal duplication cyst | Journal of Pediatric Surgery Case Reports | Ectopic Ovaries |
| Esposito | 2019 | Laparoscopic Treatment of Inguinal Ovarian Hernia in Female Infants and Children: Standardizing the Technique | Journal of Laparoendoscopic & Advanced Surgical Techniques | Ectopic Ovaries |
| Evans | 1925 | Ectopic ovary | British Journal of Surgery | Ectopic Ovaries |
| Fatora | 1974 | UNIQUE MIXOPLOID VARIANT OF OVARIAN AGENESIS | American Journal Of Human Genetics | Ineligible Patient Population |
| Fedele | 1990 | Magnetic resonance imaging in Mayer-Rokitansky-Kuster-Hauser syndrome | Obstetrics and Gynecology | Ineligible Patient Population |
| Fedele | 2007 | Laparoscopic findings and pelvic anatomy in Mayer-Rokitansky-Kuster- Hauser syndrome | Obstetrics and Gynecology | Ineligible Patient Population |
| FeiNgu | 2011 | Torsion of a tumor within an accessory ovary | Obstetrics & Gynecology | Ectopic Ovaries |
| Frank | 1909 | Papillary cystadenoma in a supernumerary ovary | Surgery Gynecology & Obstetrics | Ectopic Ovaries |
| Fregoso-Garcia | 2015 | Serous cystadenoma in supernumerary ovary. Case report and literature review. [Spanish] | GinecologÃ­a y Obstetricia de MÃ©xico | Ectopic Ovaries |
| Fruzzetti | 2015 | Unilateral adnexal agenesis and dermoid cyst: Fertility implications | Gynecological Endocrinology | Ectopic Ovaries |
| Fryssira | 2017 | Cantu Syndrome Associated with Ovarian Agenesis | Molecular Syndromology | Ineligible Patient Population |
| Fujimoto | 2019 | Incidental Finding of an Accessory Ovary at Laparoscopic Surgery | Gynecol Minim Invasive Ther | Ectopic Ovaries |
| Fujiwara | 1999 | Supernumerary ovary found by ultrasonogram and FSH measurement after an extensive operation for a yolk sac tumor of the ovary | Gynecologic and Obstetric Investigation | Ineligible Patient Population |
| Gabbay-Moore | 1982 | Accessory ovaries with bilateral dermoid cysts | European Journal of Obstetrics, Gynecology, & Reproductive Biology | Ectopic Ovaries |
| Gabriel | 2002 | Unruptured pregnancy in a non-communicating heterotopic right fallopian tube associated with left unicornuate uterus: Evidence for transperitoneal sperm and oocyte migration | Acta Obstetricia et Gynecologica Scandinavica | Ectopic Ovaries |
| GadAlRab | 2015 | Reproductive outcome re-evaluation for women with primary ovarian insufficiency using office microlaparoscopy | International Journal of Gynecology and Obstetrics | Insufficient Information to Assess Ovarian Abnormality |
| Gambadauro | 2005 | Spontaneous pregnancy following multiple laparoscopic myomectomy in a sterile patient with unilateral ovarian agenesis [6] | European Journal of Obstetrics and Gynecology and Reproductive Biology | Ectopic Ovaries |
| Garaulet | 2004 | Unilateral tuboovarian agenesis and calcified intraperitoneal mass. [Spanish] | Ciencia Ginecologika | Ectopic Ovaries |
| Garg | 2016 | Bilateral ovarian maldescent: Unusual cause of infertility - A case report and literature review | Journal of Obstetrics and Gynaecology Research | Ectopic Ovaries |
| Gaur | 2021 | The Third Ovary‑Superfuous Ovary | Journal of Obstetrics and Gynecology of India | Ectopic Ovaries |
| Georgy | 1974 | Absence of an ovary and uterine tube | Obstetrics and Gynecology | Ectopic Ovaries |
| Gheonea | 2016 | A rare case of ovarian splenosis | Rom J Morphol Embryol | Ineligible Patient Population |
| Ghirardini | 1982 | Unicorneal uterus with normal outer surface and unilateral agenesis of ovary and kidney. [German] | Zentralblatt fur GynÃ¤kologie | Non-english language |
| Gold | 1997 | Bilateral absence of the ovaries and distal fallopian tubes: A case report | Journal of Reproductive Medicine for the Obstetrician and Gynecologist | Ectopic Ovaries |
| Goldberg | 1947 | Three unusual endocrinopathies with associated ovarian pathology; ovarian agenesis; precocious puberty; virilism | Journal of Clinical Endocrinology and Metabolism | Ineligible Patient Population |
| GomezArzapalo | 1974 | Supernumerary ovary. Report of a case with cytogenetic study. [Spanish] | Ginecologia y Obstetricia de Mexico | Ectopic Ovaries |
| Gonza¡lez | 1999 | Prenatal diagnosis of ovarian cysts. The ultrasonographic course and therapeutic importance | Cirug­a pediaitrica | Ectopic Ovaries |
| GonzalezRamos | 1996 | Ipsilateral ovary and salpinx agenesis. [Spanish] | Gine-Dips | Non-english language |
| Goodman | 1988 | Absence of a vagina and right sided adnexa uteri in the Waardenburg syndrome: A possible clue to the embryological defect | Journal of Medical Genetics | Ectopic Ovaries |
| Gorgen | 2002 | Undescended fallopian tubes and ovaries: A rare incidental finding during an infertility investigation work up | Acta Obstetricia et Gynecologica Scandinavica | Ectopic Ovaries |
| Gorgojo | 2002 | Gonadal agenesis 46,XX associated with the atypical form of Rokitansky syndrome | Fertility and Sterility | Ovarian Anomaly Not Determined |
| Gorlin | 1971 | The Leopard (multiple lentigines) syndrome revisited | Birth Defects: Original Article Series | Ineligible Patient Population |
| Gotoh | 1992 | A case of primary retroperitoneal mucinous cystadenocarcinoma | Acta medica Okayama | Ectopic Ovaries |
| Gotti | 2008 | Agenesia or monolateral absence of Falloppian tube? A case report. [Italian] | Italian Journal of Gynaecology and Obstetrics | Non-english language |
| Graber | 1937 | An article on the question of ovarian aplasia with special consideration for the case of mutual absence of ovaries in newborns | Virchows Archiv far Pathologische Anatomie und Physiologie und far Klinische Medizin | Non-english language |
| Graubard | 1987 | Unilateral Mullerian and ovarian agenesis | South African medical journal = Suid-Afrikaanse tydskrif vir geneeskunde | Ectopic Ovaries |
| Grigoras | 2015 | Unilateral ovarian agenesis with partial ipsilateral tubal agenesis | Journal of Obstetrics and Gynaecology | Ectopic Ovaries |
| Grigoriadis | 2018 | Surgical management of complex atypical endometrial hyperplasia in a woman with rare genitourinary anomalies: unicornuate uterus with rudimentary horn, ipsilateral ectopic ovary and pelvic kidney | Il Giornale di chirurgia | Ectopic Ovaries |
| Grio | 1988 | A case of supernumerary ovaries diagnosed during cesarean section. [Italian] | Minerva Ginecologica | Ectopic Ovaries |
| Guadagno | 1968 | [On a case of bilateral total gonado-mullerian agenesis associated with left renal agenesis and ectopy and dysmorphism of the right kidney] | Rivista Critica di Clinica Medica | Ectopic Ovaries |
| Guileyardo | 1982 | Neonatal ovarian torsion | American Journal of Diseases of Children | Ineligible Patient Population |
| Gupta | 2016 | Mature Teratoma in a Supernumerary Ovary in a Child: Report of the First Case | Journal of Pediatric and Adolescent Gynecology | Ectopic Ovaries |
| Gupta | 2018 | Unilateral tubo-ovarian agenesis with contralateral adnexal torsion in a premenarchal girl | Case Reports | Ectopic Ovaries |
| Gupta | 2018 | Unilateral tubo-ovarian agenesis with contralateral adnexal torsion in a premenarchal girl | BMJ Case Reports | Ectopic Ovaries |
| Gursoy | 2013 | Incidental diagnosis of unilateral renal and adnexal agenesis in a 46-year-old multiparous woman | American Journal of Case Reports | Ectopic Ovaries |
| Gurumurthy | 2019 | Accessory ovary: A rare case report | Indian journal of pathology & microbiology | Ectopic Ovaries |
| Guven | 2019 | Etiologic classification of 46, XX disorders of sexual differentiation according to Chicago consensus: Single center results | Hormone Research in Paediatrics | Ineligible Patient Population |
| Guvenc | 2009 | Management of ovarian cysts during infancy: autoamputation presenting as a possible pitfall | BMJ Case Reports | Ectopic Ovaries |
| Gweon | 2016 | A successful laparoscopic neovaginoplasty using peritoneum in Mullerian agenesis with inguinal ovaries accompanied by primary ovarian insufficiency | Obstetrics & Gynecology Science | Ectopic Ovaries |
| Gyn | 2019 | Type-2 Mayer-Rokitansky-Kuster-Hauser Syndrome with Periureteric Fibrosis: A Rare Association | Pan | Ectopic Ovaries |
| Hahn-Pedersen | 1984 | Supernumerary ovary | Acta Obstetricia et Gynecologica Scandinavica | Ectopic Ovaries |
| Hall-Craggs | 2013 | Mayer-Rokitansky-Kuster-Hauser syndrome: Diagnosis with MR imaging | Radiology | Ovarian Anomaly Not Determined |
| Harlass | 1987 | Supernumerary ovary. A case report | Journal of Reproductive Medicine for the Obstetrician and Gynecologist | Ectopic Ovaries |
| Hartigan | 2006 | Intrarenal supernumerary ovary excised with partial nephrectomy | Urology | Ectopic Ovaries |
| Haydardedeoglu | 2006 | A case of unicornuate uterus with ipsilateral ovarian and renal agenesis | Fertility and Sterility | Ectopic Ovaries |
| Hayes | 1973 | ANDROBLASTOMA OF OVARY WITH HETEROTOPIC ELEMENTS | Journal of Pathology | Ectopic Ovaries |
| Heimbigner | 2020 | Benign brenner tumor in an ectopic ovary: A case report and review of literature | International Journal of Gynecological Pathology. | Ectopic Ovaries |
| Heller | 1990 | Neoplasms arising in ectopic ovaries: A case of brenner tumor in an accessory ovary | International Journal of Gynecological Pathology | Ectopic Ovaries |
| Helvacioglu | 2010 | Ovarian malposition - Mullerian anomalies revisited | Middle East Fertility Society Journal | Ectopic Ovaries |
| Herlin | 2019 | Whole-exome sequencing identifies a GREB1L variant in a three-generation family with Mullerian and renal agenesis: a novel candidate gene in Mayer Rokitansky Hauser (MRKH) syndrome. A case report | Human Reproduction | Ineligible Patient Population |
| Hertz | 1950 | A case of ovarian agenesis with normal urinary gonadotropin titer | Journal of Clinical Endocrinology and Metabolism | Ineligible Patient Population |
| Hinckley | 2003 | Percutaneous oocyte retrieval from an inguinal ovary | Fertility and Sterility | Ectopic Ovaries |
| Hogan | 1967 | Dermoid cyst in supernumerary ovary of the greater omentum. Report of a case | Obstetrics & Gynecology | Ectopic Ovaries |
| Hollander | 1967 | [Ovarian agenesis, pituitary chromophobe adenoma and Cushing's disease in one patient] | Orvosi Hetilap | Ineligible Patient Population |
| Hollander | 2008 | Unilateral renal agenesis and associated MÃ¼llerian anomalies: a case report and recommendations for pre-adolescent screening | Journal of Pediatric and Adolescent Gynecology | Ineligible Patient Population |
| Holmer | 1958 | [Implantation of embryonic ovarian tissue in cases of gonadal agenesis] | Geburtshilfe und Frauenheilkunde | Ineligible Patient Population |
| Holmer | 1948 | Girls with vestigial ovaries; agenesis ovariorum | Ned Tijdschr Geneeskd | Ectopic Ovaries |
| Hoo | 2016 | Rare Case of Leiomyoma and Adenomyosis in Mayer-Rokitansky-Kuster-Hauser Syndrome | Case Reports in Obstetrics and Gynecology | Ineligible Patient Population |
| Hughes | 2015 | Laparoscopic experience of diagnosis and removal of an atypical right iliac fossa mass | Surgical Endoscopy and Other Interventional Techniques | Ectopic Ovaries |
| Huhn | 1975 | Dermoid cysts of the greater omentum. [German] | Archiv fur Gynakologie | Ectopic Ovaries |
| Ianeva | 1973 | [Unusual case of unilateral ovarian agenesis associated with dermoid cyst] | Akusherstvo i Ginekologiia | Ectopic Ovaries |
| Idan | 2013 | Hormonal therapy in a patient with ovarian agenesis and possible SLE: A choice to be made | Clinical Rheumatology | Ovarian Anomaly Not Determined |
| Idan | 2010 | Hormonal therapy in a patient with ovarian agenesis and possible SLE: a choice to be made | Clinical Rheumatology | Ovarian Anomaly Not Determined |
| Idil | 2006 | Detection of an inguinal ovary at controlled ovarian stimulation that was successfully treated by repositioning | Fertility and Sterility | Ectopic Ovaries |
| Ihentuge |  | Ovarian agenesis associated with a hypertrophied and incompletely descended left ovary |  | Ectopic Ovaries |
| Imir | 2006 | Supernumerary ovary on sigmoid colon resembling an endometriotic lesion | Journal of Obstetrics and Gynaecology Research | Ectopic Ovaries |
| Ipek | 2010 | Prenatal over cyst torsion: US and MR imaging findings: Case report | Turkiye Klinikleri Jinekoloji Obstetrik | Ineligible Patient Population |
| Ireo | 2018 | Laparoscopic management of maldescended ovary presenting with recurrent acute abdomen | Gynecology and Minimally Invasive Therapy | Ectopic Ovaries |
| Ishimaru | 2021 | Ectopic ovary presenting as mesenteric abscess | Annals of the Royal College of Surgeons of England | Ectopic Ovaries |
| Jackson | 2015 | Adnexal Incarceration in a Posterior Pelvic Peritoneal Defect Mimics Ovarian Torsion | Journal of Minimally Invasive Gynecology | Ineligible Patient Population |
| Jang | 2020 | A case report of ectopic pregnancy arising in a unicornuate uterus, accompanied by the undescended tube and ovary with double inferior vena cava | Medicine | Ectopic Ovaries |
| Jedrzejewski | 2019 | Nuck Canal Hernias, Typical and Unusual Ultrasound Findings | Ultrasound Quarterly | Ectopic Ovaries |
| Jha | 2019 | Coexistence of gonadal dysgenesis and mullerian agenesis in a female with 46,xx karyotype: A case report | Journal of the Nepal Medical Association | Ovarian Anomaly Not Determined |
| John | 2017 | Ectopic ovary with dermoid cyst as a result of possible asymptomatic autoamputation | Journal of Human Reproductive Sciences | Ectopic Ovaries |
| Johnston | 2007 | Unilateral pelvic endometriosis and congenital unilateral ovarian agenesis [10] | Pathology | Ectopic Ovaries |
| Kadoch | 2009 | Successful pregnancy in an ovarian agenesis patient after modified natural cycle IVF oocyte donation | Reproductive BioMedicine Online | Ineligible Patient Population |
| Kakuda | 2015 | A Case of Extragonadal Teratoma in the Pouch of Douglas and Literature Review | Journal of Minimally Invasive Gynecology | Ectopic Ovaries |
| Kamar | 2020 | Case Report: Unilateral Ovarian Absence (UOA) with Unilateral Ovarian Hypoplasia and Uterine Hypoplasia | J Nurs Occup Health | Ectopic Ovaries |
| Kamiyama | 2001 | Two cases of supernumerary ovary: One with large fibroma with Meig's syndrome and the other with endometriosis and cystic change | Pathology Research and Practice | Ectopic Ovaries |
| Kanter | 2017 | Management of ectopic ovary in the setting of mullerian agenesis | Obstetrics and Gynecology | Ectopic Ovaries |
| Kanter | 2017 | Management of ectopic ovary in the setting of mullerian agenesis [35P] | Obstetrics & Gynecology | Ectopic Ovaries |
| Kapczuk | 2016 | Congenital malformations and other comorbidities in 125 women with Mayer-Rokitansky-Kuster-Hauser syndrome | European Journal of Obstetrics and Gynecology and Reproductive Biology | Ovarian Anomaly Not Determined |
| Kara | 2013 | MRI in the diagnosis of Mayer-Rokitansky-Kuster-Hauser syndrome | Diagnostic and Interventional Radiology | Ineligible Patient Population |
| Kawasaki | 1967 | Chromosome studies of dysgenesis and agenesis ovarii | Journal of the Japanese Obstetrical and Gynecological Society | Ovarian Anomaly Not Determined |
| Kaya | 2003 | Mayer-Rokitansky-Kuster-Hauser Syndrome Associated with Unilateral Gonadal Agenesis: A Case Report | Journal of Reproductive Medicine for the Obstetrician and Gynecologist | Ectopic Ovaries |
| Kdous | 2008 | [Gonadal agenesis 46,XX associated with Mayer-Rokitansky-Kuster-Hauser syndrome. A rare association] | Tunisie Medicale | Non-english language |
| Keasling | 1959 | Incarcerated femoral hernia containing an ovary | Journal of the American Medical Association | Ectopic Ovaries |
| Keating | 1995 | Hernia uterus inguinale associated with unilateral renal agenesis | Australian and New Zealand Journal of Surgery | Ectopic Ovaries |
| Keitoku | 1997 | Extraovarian sex cord-stromal tumor: Case report and review of the literature | International Journal of Gynecological Pathology | Ectopic Ovaries |
| Kerkhof | 1955 | [Two cases of so-called ovarian hypoplasia or primary partial agenesis of ovaries] | Nederlandsch Tijdschrift voor Verloskunde en Gynaecologie | Ectopic Ovaries |
| Kerkhof | 1956 | Two cases of hypoplasia of the ovaries; partial primary agenesia of the gonads | Acta Endocrinologica | Ineligible Patient Population |
| Khan | 2021 | Mayer-Rokitansky-Küster-Hauser Syndrome: MR Manifestations Of Typical And Atypical Cases | J Ayub Med Coll Abbottabad | Ineligible Patient Population |
| Kilicdag | 2005 | Absence of unilateral adnexa associated with asymptomatic adnexal torsion and autoamputation | Gynecological Surgery | Ectopic Ovaries |
| Kim | 2020 | Ovarian dysgerminoma with Mullerian anomaly: a case report | Obstetrics & Gynecology Science | Ineligible Patient Population |
| Kim | 2014 | Complete septate uterus, obstructed hemivagina, and ipsilateral adnexal and renal agenesis in pregnancy | Obstetrics & Gynecology Science | Ectopic Ovaries |
| Kini | 1998 | Supernumerary ovary associated with Wilms' tumor | Pediatric Surgery International | Ectopic Ovaries |
| Kiuchi | 2016 | Uterine cervical adenocarcinoma metastasizing concurrently to eutopic and ectopic ovaries: A case report | Journal of Obstetrics and Gynaecology Research. | Ectopic Ovaries |
| Kives | 2004 | Ruptured hemorrhagic cyst in an undescended ovary | Journal of Pediatric Surgery | Ectopic Ovaries |
| Klan | 1987 | Supernumerary Ovary - a Rare Cystic Lesion in the Retroperitoneum | Aktuelle Urologie | Ectopic Ovaries |
| Knudsen | 1994 | An extra-uterine pregnancy in an ectopic fallopian tube | Zentralblatt fur GynÃ¤kologie | Ectopic Ovaries |
| Kolawole | 1981 | Pelvic pneumography in the investigation of patients with primary amenorrhoea | Diagnostic Imaging | Ineligible Patient Population |
| Kollia | 2014 | True ectopic ovary in the right iliac fossa mimicking acute appendicitis and associated with ipsilateral renal agenesis | Journal of Obstetrics and Gynaecology Research | Ectopic Ovaries |
| Koohmanaee | 2021 | Case Report: The Coexistence of Gonadal Dysgenesis With Mayer-rokitansky-küster-hauser Syndrome, and Dandy-Walker Variant | Caspian Journal of Neurological Sciences | Ineligible Patient Population |
| Kosasa | 1976 | Diagnosis of a supernumerary ovary with human chorionic gonadotropin | Obstetrics and Gynecology | Ectopic Ovaries |
| Koura | 2017 | Unicornuate uterus with ectopic ovary: A case report | BJOG: An International Journal of Obstetrics and Gynaecology | Ectopic Ovaries |
| Kriss | 1947 | Neoplasm of a supernumerary ovary; report of two cases | Journal of the Mount Sinai Hospital, New York | Ectopic Ovaries |
| Kuga | 1999 | A supernumerary ovary of the omentum with cystic change: Report of two cases and review of the literature | Pathology International | Ectopic Ovaries |
| Kurtz | 1992 | BENIGN SEROUS CYSTADENOMA IN AN ACCESSORY OVARY DIAGNOSED AT LAPAROSCOPY - REPORT OF 2 CASES | Journal of Gynecologic Surgery | Ectopic Ovaries |
| Kutlucan | 2012 | Leopard syndrome presented with hemolytic anemia, total genital prolapse and ovarian agenesis | Duzce Medical Journal | Ineligible Patient Population |
| Lachman | 1991 | The ectopic ovary. A case report and review of the literature | Archives of Pathology and Laboratory Medicine | Ectopic Ovaries |
| Laing | 2007 | Ovary-containing hernia in a premature infant: sonographic diagnosis | Journal of Ultrasound in Medicine | Ectopic Ovaries |
| Lam | 1987 | Outcome of patients with one ovary in an in vitro fertilization program | Journal of in Vitro Fertilization and Embryo Transfer | Ovarian Anomaly Not Determined |
| Lara-Torre | 2005 | Mullerian agenesis and ovarian torsion. a case report and review of literature | Journal of Pediatric Surgery | Ectopic Ovaries |
| Laroche | 1958 | [Case of ovarian agenesis without nanism & with normal feminine morphology] | Bulletins et Mémoires de la Société Médicale des Hôpitaux de Paris | Ineligible Patient Population |
| Laroche | 1964 | [Isolated Ovarian Agenesis with Normal Physique] | Annales d Endocrinologie | Ineligible Patient Population |
| Lee | 1984 | A case of supernumerary ovary | Obstetrics and Gynecology | Ectopic Ovaries |
| Lee | 2019 | True Ectopic Ovary with Mature Cystic Teratoma | Journal of Minimally Invasive Gynecology | Ectopic Ovaries |
| Leiva | 2021 | The Association Between Ovarian Maldescent and Uterine Septum | J. Gynecol. Surg. | Ectopic Ovaries |
| Levavi | 1996 | Recurrent bilateral dermoid cysts in accessory ovaries | Acta Obstet Gynecol Scand | Ectopic Ovaries |
| Levi | 1952 | Malignant melanoma in a patient with ovarian agenesis; case report of prolonged survival | Journal of Clinical Endocrinology and Metabolism | Ovarian Anomaly Not Determined |
| Levy | 1997 | Intrarenal supernumerary ovary | Journal of Urology | Ectopic Ovaries |
| Liaqat | 2021 | Auto-amputated ovary- rare cause of intestinal obstruction in a neonate: A case report | Journal of Neonatal Surgery | Ineligible Patient Population |
| Lim | 2004 | Two dermoid cysts developing in an accessory ovary and an eutopic ovary | Journal of Korean Medical Science | Ectopic Ovaries |
| Lim | 2018 | Supernumerary ovary on recto-sigmoid colon with associated endometriosis | Obstetrics & Gynecology Science | Ectopic Ovaries |
| Lisser | 1946 | THE SYNDROME OF CONGENITALLY ABSENT OVARIES, WITH INFANTILISM, HIGH URINARY GONADOTROPINS AND SHORT STATURE, WITH OTHER CONGENITAL ABNORMALITIES, SUCH AS SHORT WEBBED NECK, CUBITUS VALGUS, COARCTATION OF THE AORTA, ETC., AND TABULAR PRESENTATION OF 21 PRE | Journal of Clinical Endocrinology & Metabolism | Duplicate Study Data |
| Lisser | 1946 | THE SYNDROME OF CONGENITALLY ABSENT OVARIES, WITH INFANTILISM HIGH URINARY GONADOTROPINS AND SHORT STATURE, WITH OTHER CONGENITAL ABNORMALITIES, SUCH AS SHORT WEBBED NECK, CUBITUS VALGUS COARCTATION OF THE AORTA, ETC, AND TABULAR PRESENTATION OF 21 PREVIO | Endocrinology | Conference Abstract |
| Litos | 2003 | Supernumerary ovary: A case report and literature review | Journal of Obstetrics and Gynaecology | Ectopic Ovaries |
| Liu | 2005 | Steroid cell tumors, not otherwise specified (NOS), in an accessory ovary: a case report and literature review | Gynecol Oncol | Ectopic Ovaries |
| Louda | 2012 | Perrault's syndrome: A case report | Medecine Therapeutique Medecine de la Reproduction, Gynecologie et Endocrinologie | Ineligible Patient Population |
| Luvero | 2017 | Undescended or absent ovary without uterine anomalies: Systematic review and a single center's experience | Minerva Ginecologica | Duplicate |
| Machicls | 1998 | Unusual location of an ovary: Ultrasonographic features and surgical correlation | Journal Belge de Radiologie | Ectopic Ovaries |
| Mahmoud | 2014 | Maldescended ovary associated with Mullerian and kidney dysgenesis: a case report | Journal of Reproductive Medicine | Ectopic Ovaries |
| Mama | 2012 | Ectopic ovary presenting as right upper quadrant abdominal pain in a premenarchal patient with a unicornuate uterus | Journal of Pediatric and Adolescent Gynecology | Ectopic Ovaries |
| Matsubara | 2009 | Periodic size changes in a supernumerary ovary with associated corpus luteal cyst | Journal of Obstetrics and Gynaecology Research | Ectopic Ovaries |
| Matthews | 2014 | Diagnosis and management of an ovarian cyst complicated by torsion in utero: A case report | Journal of Pediatric Surgery Case Reports | Ineligible Patient Population |
| Matyakhina | 2007 | Discordance for ovarian dysgenesis in a pair of monozygotic twins | Endocrinologist | Ineligible Patient Population |
| MasoumiShahrbabak | 2021 | A rare case of Mayer-Rokitansky-Küster-Hauser syndrome with right ovarian torsion and hypoplasia of the left adnexa | J Surg Case Rep | Ineligible Patient Population |
| McCullough | 1992 | Supernumerary or ectopic ovary: A case report | Histopathology | Ectopic Ovaries |
| Mendez | 1986 | Endocrine evaluation in a patient with MURCS association and ovarian agenesis | European Journal of Obstetrics Gynecology and Reproductive Biology | Ineligible Patient Population |
| Mendonca | 1994 | Gonadal agenesis in XX and XY sisters: Evidence for the involvement of an autosomal gene | American Journal of Medical Genetics | Ovarian Anomaly Not Determined |
| Mercer | 1987 | Tumors originating in supernumerary ovaries. A report of two cases | Journal of Reproductive Medicine for the Obstetrician and Gynecologist | Ectopic Ovaries |
| MilnesWalker | 1935 | UNDESCENDED OVARIES | The Lancet | Ectopic Ovaries |
| MilnesWalker | 1933 | A CASE OF UNDESCENDED OVARY | The Lancet | Duplicate |
| Min | 2011 | Ectopic Ovary at Ectopic Pregnancy | Journal of Minimally Invasive Gynecology | Ectopic Ovaries |
| Miolo | 2016 | Gonadal agenesis with hypoplastic paramesonephric ducts (PMNDs) derivatives in dizygotic twins | Gynecological Endocrinology | Ovarian Anomaly Not Determined |
| Mirowski | 1964 | Right ventricular aneurysm, a complication of transventricular pulmonary valvulotomy. Report of two cases, one associated with gonadal agenesis | American Heart Journal | Ineligible Patient Population |
| Mittal | 2013 | Ectopic ovarian leiomyoma presenting as chronic pelvic pain | Journal of Gynecologic Surgery | Ectopic Ovaries |
| Mittal | 2011 | A rare case of undescended left uterine horn | Gynecological Surgery | Ectopic Ovaries |
| Miura | 2016 | Dysgerminoma developing from an ectopic ovary in a patient with WAGR syndrome: A case report | Molecular and Clinical Oncology | Ectopic Ovaries |
| Mochizuki | 2006 | [Abnormal gonadal development] | Nippon Rinsho - Japanese Journal of Clinical Medicine | No Original Data |
| Mohanty | 2017 | A rare case of adult ovarian hernia in MRKH syndrome | BJR Case Reports | Ectopic Ovaries |
| Mooren | 2021 | A Retrospective Analysis of Female Mullerian Duct Anomalies in Association With Congenital Renal Abnormalities | Journal of Pediatric and Adolescent Gynecology | Ineligible Patient Population |
| Moncreiffe | 2014 | Congenitally absent unilateral adnexa with a large paratubal cyst | Gynecological Surgery | Conference Abstracts |
| Monrozies | 1971 | [A case of ovarian agenesis. Clinical and cytogenetical study] | Bulletin de la Federation des Societes de Gynecologie et d Obstetrique de Langue Francaise | Ineligible Patient Population |
| Monteagudo | 2010 | Congenital subcostal hernia with unusual contents | Journal of Pediatric Surgery | Ectopic Ovaries |
| Mora | 2021 | 56. Rare Presentation of Mullerian Duct Anomaly | Journal of Pediatric and Adolescent Gynecology | Ectopic Ovaries |
| Morabito | 2020 | A young girl with right ovarian torsion and left ovarian ectopy | Italian Journal of Pediatrics | Duplicate |
| Morabito | 2020 | A young girl with right ovarian torsion and left ovarian ectopy | Italian Journal of Pediatrics | Ectopic Ovaries |
| Moustafa | 2001 | Benign cystic teratoma in an ectopic ovary | Journal of Obstetrics and Gynaecology | Ectopic Ovaries |
| Movilla | 2019 | Prepared for the Unexpected: Accessory Ovaries with Abernethy Malformation | Journal of Minimally Invasive Gynecology | Ectopic Ovaries |
| MraniAlaoui | 2014 | SFCP P-049 - Neonatal ovarian torsion: About 2 observations. [French] | Archives de Pediatrie | Ineligible Patient Population |
| Mulayim | 2003 | Unicornuate uterus and unilateral ovarian agenesis associated with pelvic kidney | Surgical Endoscopy | Conference Abstract |
| Mullan | 1994 | Ectopic fallopian tube and ovarian cystadenocarcinoma in an inguinal hernia | European Journal of Surgery, Acta Chirurgica | Ectopic Ovaries |
| Nandy | 2019 | Ovarian agenesis and Mullerian duct dysgenesis in a karyotypically normal (46, XX) pre-pubertal girl with aberrant cognition: A case report and literature review | International Journal of Medical Reviews and Case Reports | Ineligible Patient Population |
| Navarro | 1990 | Supernumerary ovary in association with endometriosis | Fertility and Sterility | Ectopic Ovaries |
| New | 2018 | Adnexal torsion in a patient with MÃ¼llerian agenesis undergoing ovarian stimulation: a case report | Proceedings in Obstetrics and Gynecology | Ineligible Patient Population |
| Nichols | 2009 | Case of Accessory Ovary in the Round Ligament with Associated Endometriosis | Journal of Minimally Invasive Gynecology | Ectopic Ovaries |
| Nichols | 1951 | Congenital ectopic ovary | American Journal of Obstetrics and Gynecology | Ectopic Ovaries |
| Nickles | 2012 | All is not as it seems: Bleeding through several diagnoses in a perimenarchal girl | Journal of Pediatric and Adolescent Gynecology | Ectopic Ovaries |
| Nigam | 2010 | Septate uterus with hypoplastic left adnexa with cervical duplication and longitudinal vaginal septum: Rare Mullerian anomaly | Journal of Human Reproductive Sciences | Ineligible Patient Population |
| Nishio | 2011 | Two cases of ectopic ovary and one case of potential ectopic ovary | Reproductive Medicine and Biology | Ectopic Ovaries |
| Nishiyama | 2010 | Bilateral Interruption of Mid-Fallopian Tubes and Ovarian Anomalies Including Ectopic Ovary and Cystic Teratoma, a Previously Unreported Combination | Journal of Minimally Invasive Gynecology | Ectopic Ovaries |
| Nomelini | 2013 | Serous papillary cystadenocarcinoma in supernumerary ovary | Journal of Obstetrics and Gynaecology | Ectopic Ovaries |
| Ogishima | 2017 | Cystic Endometrioma with Coexisting Fibroma Originating in a Supernumerary Ovary in the Rectovaginal Pouch | Case Reports in Obstetrics and Gynecology | Ectopic Ovaries |
| Oliveira | 2014 | Ectopic ovary after previous bilateral oophorectomy in a breast cancer patient | Journal of Obstetrics and Gynaecology | Ectopic Ovaries |
| Ombelet | 2003 | Undescended ovary and unicornuate uterus: Simplified diagnosis by the use of clomiphene citrate ovarian stimulation and magnetic resonance imaging (MRI) (vol 18, pg 858, 2003) | Human Reproduction | Ectopic Ovaries |
| Ombelet | 2003 | Intrauterine pregnancy following transperitoneal oocyte and/or sperm migration in a woman with an ectopic (undescended) ovary | Reproductive BioMedicine Online | Ectopic Ovaries |
| Ombelet | 2003 | Undescended ovary and unicornuate uterus: Simplified diagnosis by the use of clomiphene citrate ovarian stimulation and magnetic resonance imaging (MRI) | Human Reproduction | Ectopic Ovaries |
| Ombelet | 2003 | Ectopic ovary and unicornuate uterus [7] | New England Journal of Medicine | Ectopic Ovaries |
| Ombelet | 2011 | Unicornuate uterus and ectopic (undescended) ovary | Facts Views & Vision in Obgyn | Ectopic Ovaries |
| Omori | 2017 | Extraovarian Fibroma with Minor Sex Cord Elements: A Case Report and Literature Review | International Journal of Surgical Pathology | Ectopic Ovaries |
| Omurtag | 2011 | The autoamputated adnexae: examples of common presentations | Journal of Pediatric and Adolescent Gynecology | Conference Abstract |
| Opdecam | 2021 | Misdiagnosis of associated mullerian agenesis in a female with 46, XX gonadal dysgenesis: a case report and review of literature | J Obstet Gynaecol | Ineligible Patient Population |
| Oride | 2016 | Rare case of complicated congenital anomalies of female reproductive organs with bilateral undescended ovaries | Journal of Obstetrics and Gynaecology Research. | Ectopic Ovaries |
| Otjen | 2015 | A normal ovary in an abnormal location: A case of torsion | Journal of clinical ultrasound : JCU | Ectopic Ovaries |
| Ozcan | 2015 | Imaging Findings of Fetal-Neonatal Ovarian Cysts Complicated With Ovarian Torsion and Autoamputation | American Journal of Roentgenology | Ectopic Ovaries |
| Ozyazgan | 2018 | Use of infragluteal folds as a full-thickness skin graft donor site for construction of the neovagina in cases of Mullerian agenesis | Journal of Obstetrics and Gynaecology Research | Insufficient Information to Assess Ovarian Abnormality |
| Pampal | 2013 | Torsion of the Ovary in an Incarcerated Inguinal Hernia | Pediatric Emergency Care | Ectopic Ovaries |
| Pan | 2019 | A Rare Ectopic Ovary Mimicking Colon Sigmoideum Mesenchymoma Presenting as an Intestinal Mesenchymoma | Frontiers in Oncology | Ectopic Ovaries |
| Panoskaltsis | 2001 | Mucinous cystadenocarcinoma in an ectopic, retroperitoneal ovary | Journal of Obstetrics and Gynaecology | Ectopic Ovaries |
| Park | 2015 | Rare Case of Cystadenoma From an Ectopic Ovary Presenting With Acute Abdominal Pain | Journal of Minimally Invasive Gynecology | Ectopic Ovaries |
| Paul | 2020 | Congenital uterovaginal abnormalities, it's embryogenesis, surgical management and clinical implications | Obstetrics & Gynecology Science | Ineligible Patient Population |
| Pearl | 1963 | Supernumerary ovary. Report of a case | Obstetrics & Gynecology | Ectopic Ovaries |
| Peedicayil | 1992 | Ectopic ovary in the omentum | Asia-Oceania journal of obstetrics and gynaecology / AOFOG | Ectopic Ovaries |
| Peitsidou | 2009 | Diagnosis of an autoamputated ovary with dermoid cyst during a Cesarean section | Fertility and Sterility | Ineligible Patient Population |
| Pennell | 1989 | Retroperitoneal mucinous cystadenoma | American Journal of Obstetrics and Gynecology | Ineligible Patient Population |
| Pereira | 2021 | Combined transvaginal and transabdominal oocyte retrieval in a patient with an ectopic ovary and unicornuate uterus | Fertility and Sterility | Ectopic Ovaries |
| Petersen | 1982 | Anal atresia combined with unilateral agenesis of the ovary and the uterine tube. [Danish] | Ugeskrift for Laeger | Non-english language |
| Pfitzmann | 2004 | A dermoid cyst in the greater omentum as a rare epigastric tumor | Zeitschrift Fur Gastroenterologie | Ectopic Ovaries |
| Philipp | 1951 | Clinical aspects of gonadal agenesia | Die Medizinische Welt | Duplicate |
| Philipp | 1952 | Clinical aspects of ovarian agenesia | El DÃ­a mÃ©dico | Non-english language |
| Philipp | 1951 | Clinical picture of congenital absence of the genital glands | Gynecologie Pratique | Non-english language |
| Pineda | 2006 | Unilateral ovarian agenesis: Usefulness of hysterosalpingography for etiological diagnosis. A case report | Progresos en Obstetricia y Ginecologia | Ectopic Ovaries |
| Pittock | 2005 | Mayer-Rokitansky-Kuster-Hauser anomaly and its associated malformations | American Journal of Medical Genetics | Insufficient Information to Assess Ovarian Abnormality |
| Poma | 1982 | Supernumerary ovary | Imj | Ectopic Ovaries |
| Popatia | 2013 | Utility of exercise flow-volume loops in an unusual presentation of vascular ring | American Journal of Respiratory and Critical Care Medicine. Conference: American Thoracic Society International Conference, ATS | Ovarian Anomaly Not Determined |
| Popovic | 1973 | Malformations of the genitals: the cause of some primary amenorrheas | ARCH.UN.MED.BALKAN | Non-english language |
| Praest | 1976 | Uterus unicornis. Report of a case with simultaneous agenesis of the contralateral kidney, ovary and adnexa (Danish). [Danish] | Ugeskrift for Laeger | Non-english language |
| Prakash | 2016 | Laparoscopic management of tumor in supernumerary ovary | Journal of Mid-life Health | Ectopic Ovaries |
| Printz | 1973 | The embryology of supernumerary ovaries | Obstetrics & Gynecology | Ectopic Ovaries |
| Pujman | 1949 | [Cancer of a supernumerary ovary] | Casopis Lekaru Ceskych | Ectopic Ovaries |
| Radwin | 1949 | PRIMARY OVARIAN AGENESIS | Journal of Pediatrics | Ovarian Anomaly Not Determined |
| Ramage | 1896 | CONGENITAL ABSENCE OF THE OVARIES WITH RUDIMENTARY UTERUS | The Lancet | Ectopic Ovaries |
| Randerath | 1925 | On a case of the congenital absence of both ovaries | Virchows Archiv fur Pathologische Anatomie und Physiologie und fÃ¼r Klinische Medizin | Non-english language |
| Reed | 1990 | Ectopic ovaries associated with absent uterus and pelvic kidney: CT findings | Journal of Computer Assisted Tomography | Ectopic Ovaries |
| Reinhold | 1997 | Primary amenorrhea: Evaluation with MR imaging | Radiology | Ineligible Patient Population |
| Rodriguez-Ruiz | 2020 | Supernumerary ovary in a pregnant patient. Case report and literature review. [Spanish] | Ginecologia y Obstetricia de Mexico | Ectopic Ovaries |
| Ross | 1968 | Incidental finding of an accessory ovary | Am J Obstet Gynecol | Ectopic Ovaries |
| Rossle | 1930 | Congenital absence of the ovaries and its fundamental significance for the theory of the determination of gender | Beitrage Zur Pathologischen Anatomie Und Zur Allgemeinen Pathologie | Non-english language |
| Roth | 1996 | Steroid cell tumor of the broad ligament arising in an accessory ovary | Archives of Pathology & Laboratory Medicine | Ectopic Ovaries |
| Rowley | 1948 | Uterine anomaly; duplication of uterus, three tubes and three ovaries; report of a case | Annals of Surgery | Ectopic Ovaries |
| Saini | 2021 | Ovarian inguinal hernia - a possibility in MURCS syndrome | Journal of Ovarian Research | Ectopic Ovaries |
| Sanders | 1928 | Congenital absence of one ovary and the corresponding fallopian tube | British Medical Journal | Ectopic Ovaries |
| Sasaki | 2019 | Left ovarian transposition of undescended ovary with unicornuate uterus | Fertility and Sterility | Ectopic Ovaries |
| Sasaki | 2019 | Left Ovarian Transposition of Undescended Ovary with Unicornuate Uterus | Journal of Minimally Invasive Gynecology | Ectopic Ovaries |
| Sasano | 1963 | Congenital Adrenal Hyperplasia Associated with Gonadal Agenesis | Endocrinologia Japonica | Ineligible Patient Population |
| Scalia | 1984 | Benign teratoma in supernumerary ovary. [Italian] | Pathologica | Ectopic Ovaries |
| Schneider | 1955 | Heterotopic bone in the ovary | Ochsner Clinic reports | Ineligible Patient Population |
| Sekmenli | 2017 | Ruptured hemorrhagic cyst of undescended ovary mimicking mucocele: A rare pediatric case | Iranian Journal of Medical Sciences | Ectopic Ovaries |
| Senechal | 1948 | On a case of tubo-ovarian anomaly | Comptes Rendus de la Societe Francaise de Gynecologie (1931) | Non-english language |
| Serment | 1964 | [Ovarian Agenesis and Hyperplasia of the Cells of the Hilum] | Bulletin de la Federation des Societes de Gynecologie et d Obstetrique de Langue Francaise | Non-english language |
| Sharatz | 2008 | Giant serous cystadenoma arising from an accessory ovary in a morbidly obese 11-year-old girl: A case report | Journal of Medical Case Reports | Ectopic Ovaries |
| Shetty | 2013 | Unreported location and presentation for a parasitic ovarian dermoid cyst in an indirect inguinal hernia | Hernia: The Journal of Hernias and Abdominal Wall Surgery | Ectopic Ovaries |
| Shih | 2015 | Undescended ovary mimicking appendiceal mucocele | Surgery (United States) | Ectopic Ovaries |
| Siam | 2014 | Combined laparoscopy and hysteroscopy for the detection of female genital system anomalies: Results of 3,811 infertile women | Journal of Reproductive Medicine | Ectopic Ovaries |
| Silver | 1954 | Ovarian agenesis (congenital aplastic ovaries) in children | Obstetrical and Gynecological Survey | Ineligible Patient Population |
| Sinonquel | 2018 | Undescended ovary and fallopian tube presenting as appendiceal mucocele | Facts Views & Vision in Obgyn | Ectopic Ovaries |
| Skjelbred | 1953 | Ovarian agenesis or the pterygium syndrome | Archives of Disease in Childhood | Ovarian Anomaly Not Determined |
| Sloan | 2006 | Mullerian agenesis and gonadal abnormality misdiagnosed as delayed puberty in a woman with Fanconi's anemia | Journal of Investigative Medicine | Conference Abstract |
| Sofia | 2012 | Women with one ovary in assisted reproduction technologies: A review of the literature | Archives of Gynecology and Obstetrics | No Original Data |
| Sonntag | 2005 | Retroperitoneal mucinous adenocarcinoma occuring during pregnancy in a supernumerary ovary | Journal of Obstetrics and Gynaecology | Ectopic Ovaries |
| Stanescu | 2016 | Neonatal and infantile ovarian torsion: A spectrum of imaging findings with pathologic correlation in a large series of patients | Pediatric Radiology | Ectopic Ovaries |
| Stefanopol | 2022 | Diagnostic and Management of Undescended Ovary - A Preoperative Dilemma: A Case-Based Systematic Review | International Journal of Women's Health | Ectopic Ovaries |
| Stephens | 1986 | The Mayer-Rokitansky syndrome | Journal of Urology | No Original Data |
| Stone | 1949 | Absence of tube and ovary, congenital or acquired | American Journal of Obstetrics and Gynecology | Ectopic Ovaries |
| Strissel | 2009 | Assessment of Pituitary and Steroid Hormones and Members of the TGF-beta Superfamily for Ovarian Function in Patients with Congenital Uterus and Vaginal Aplasia (MRKH Syndrome) | Hormone and Metabolic Research | Ovarian Anomaly Not Determined |
| Strubbe | 1993 | Mayer-Rokitansky-Kuster-Hauser syndrome: Distinction between two forms based on excretory urographic, sonographic, and laparoscopic findings | American Journal of Roentgenology | Ovarian Anomaly Not Determined |
| Stuti | 2015 | An incidental finding of unicornuate uterus with unilateral ovarian agenesis and ipsilateral twining of fallopian tubes during cesarean | JARMS | Paper could not be obtained |
| Suh | 2016 | Ruptured Hemorrhagic Corpus Luteum Cyst in an Undescended Ovary: A Rare Cause of Acute Abdomen | Journal of Pediatric and Adolescent Gynecology | Ectopic Ovaries |
| Suneja | 1996 | An unusual presentation of fallopian tubes and ovaries in inguinal canal | Journal of the Indian Medical Association | Ectopic Ovaries |
| Svokos | 2020 | Case report title information: Adnexal torsion in a premenarchal female with absence of contralateral ovary and fallopian tube | Journal of Pediatric Surgery Case Reports | Duplicate |
| Svokos | 2020 | Case report title information: Adnexal torsion in a premenarchal female with absence of contralateral ovary and fallopian tube | Journal of Pediatric Surgery Case Reports | Ectopic Ovaries |
| Tanaka | 2022 | Lobulated ovary as a rare congenital anomaly: MR findings | Radiology Case Reports | Ineligible Patient Population |
| Taner | 2010 | Ectopic ovary in a patient with endometriosis. [Turkish] | Turk Jinekoloji ve Obstetrik Dernegi Dergisi | Ectopic Ovaries |
| Temiz | 2008 | A giant serous cystadenoma developing in an accessory ovary | Archives of Gynecology and Obstetrics | Ectopic Ovaries |
| Thompson | 2012 | Ovarian torsion in childhood | Pearls and Pitfalls in Pediatric Imaging: Variants and Other Difficult Diagnoses | No Original Data |
| Thomson | 1948 | Complete congenital absence of the vagina associated with bilateral herniae of uterus, tubes, and ovaries | British Journal of Surgery | Ectopic Ovaries |
| TokerKurtmen | 2022 | The Role of Surgery in Antenatal Ovarian Torsion: Retrospective Evaluation of 28 Cases and Review of the Literature | J Pediatr Adolesc Gynecol | Ineligible Patient Population |
| Torres-Oliver | 1953 | Left inguinal hernia involving the ovary, tube and uterus with concomitant absence of the vagina; report of a case | Boletin - Asociacion Medica de Puerto Rico | Ectopic Ovaries |
| Toussi | 1980 | Renal hypodysplasia and unilateral ovarian agenesis in the penta-X syndrome | American Journal of Medical Genetics | Ineligible Patient Population |
| Trinidad | 2004 | Ovarian maldescent | European Radiology | Ectopic Ovaries |
| Trott | 1996 | Laparoscopic reduction of an ovary incarcerated in a right inguinal hernia in Mullerian agenesis | Journal of Gynecologic Surgery | Ectopic Ovaries |
| Tunney | 1950 | Inguinal ectopia of the ovary, tube and uterus; report of a case with associated tenitourinary anomalies | N Engl J Med | Ectopic Ovaries |
| Turner | 1950 | Ovarian agenesis and rudimentary ovaries | Progress in Clinical Endocrinology | No Original Data |
| Uckuyu | 2009 | Unilateral Congenital Ovarian Absence with Twisted Tube | Journal of Minimally Invasive Gynecology | Duplicate |
| Uckuyu | 2009 | Unilateral congenital ovarian and partial tubal absence: Report of four cases with review of the literature | Fertility and Sterility | Ineligible Patient Population |
| Ueda | 2016 | Right Inguinal Hernia Encompassing the Uterus, Right Ovary and Fallopian Tube in an Elderly Female: Case Report | Journal of Nippon Medical School | Ectopic Ovaries |
| Uguralp | 2007 | A torsioned and autoamputated ovarian cyst simulating a duplication cyst: A case report | Turkish Journal of Medical Sciences | Ineligible Patient Population |
| Uyar | 2011 | Ectopic ovary confirmed by ovarian stimulation in a case of unicornuate uterus | Fertility and Sterility | Ectopic Ovaries |
| Valizadeh | 2011 | A case of factor five deficiency associated with gonadal agenesis | Journal of Thrombosis and Haemostasis | Ineligible Patient Population |
| VanCreveld | 1949 | Hypoplasia or agenesis of the ovary | Ned Tijdschr Geneeskd | Non-english language |
| VanCreveld | 1949 | [A case of ovarien agenesis] | Nederlands Tijdschrift voor Geneeskunde | Non-english language |
| VanVoorhis | 2000 | Bilateral undescended ovaries: Association with infertility and treatment with IVF | Fertility and Sterility | Ectopic Ovaries |
| Vaughn | 2000 | Laparoscopic repair of bilateral inguinal hernias in a patient with mullerian agenesis | Fertility and Sterility | Ectopic Ovaries |
| Veasey | 2021 | Primary ovarian insufficiency in adolescents: Clinical presentations, aetiology and initial investigations | Australian and New Zealand Journal of Obstetrics and Gynaecology | Conference Abstract |
| Verkauf | 1996 | Ovarian maldescent | Fertility & Sterility | Ectopic Ovaries |
| Verma | 2018 | Mayer-Rockitansky-Kuster-Hauser Syndrome Presenting as Irreducible Inguinal Hernia | Indian Journal of Surgery | Ectopic Ovaries |
| Verrilli | 2020 | Surgical Management of Undescended Left Ovary and Rudimentary Uterine Horn | Fertility and Sterility | Ectopic Ovaries |
| Vontheobald | 1987 | HETEROTOPIC AUTOTRANSPLANTATION OF THE OVARY IN WOMEN | Presse Medicale | Ineligible Patient Population |
| Walker | 1935 | Undescended ovaries | Lancet | Duplicate |
| Walker | 1933 | A case of undescended ovary | Lancet | Ectopic Ovaries |
| Wang | 2020 | Typical and atypical pelvic MRI characteristics of Mayer-Rokitansky-Kuster-Hauser syndrome: a comprehensive analysis of 201 patients | European Radiology | Ectopic Ovaries |
| Wang | 2018 | Increased incidence of abnormally located ovary in patients with Mayer-Rokitansky-Kuster-Hauser syndrome: a retrospective analysis with magnetic resonance imaging | Abdominal Radiology | Ineligible Patient Population |
| Wang | 2017 | Evaluation of Mayer-Rokitansky-Kuster-Hauser syndrome with magnetic resonance imaging: Three patterns of uterine remnants and related anatomical features and clinical settings | European Radiology | Ineligible Patient Population |
| Watkins | 2004 | True ectopic ovary: A case and review | Archives of Gynecology and Obstetrics | Ectopic Ovaries |
| Webb | 2014 | The management of an ectopic ovary in the inguinal canal: literature review and discussion | Pediatric Surgery International | Ectopic Ovaries |
| Wei | 2021 | Undescended ovary without abnormal development of uterus and urinary system: a report of four cases | Journal of Ovarian Research | Ectopic Ovaries |
| Wharton | 1959 | Two cases of supernumerary ovary and one of accessory ovary, with an analysis of previously reported cases | American Journal of Obstetrics & Gynecology | Ectopic Ovaries |
| Wheeler | 1985 | Transperitoneal migration of the ovum. A case report | Journal of Reproductive Medicine for the Obstetrician and Gynecologist | Ineligible Patient Population |
| Whitaker | 1997 | Serous cystadenofibroma arising in an ectopic ovary | Kansas medicine : the journal of the Kansas Medical Society | Ectopic Ovaries |
| Wimpers | 2021 | [Inguinal hernia in children: easily incarcerated] | Ned Tijdschr Geneeskd | Non-english language |
| Wong | 2013 | Pregnancy complicated by morbidly adherent placenta in a patient with bilateral ovarian agenesis: A case report | International Journal of Women's Health | Ineligible Patient Population |
| Wozniak | 2014 | Intrahepatic ovulation | Radiology Case Reports | Ectopic Ovaries |
| Xatzipsalti | 2017 | A rare case of a girl with growth hormone deficiency and a phenotype of genetic abnormality of FSH receptor | Hormone Research in Paediatrics | Conference Abstract |
| Xu | 2011 | A case of accessory ovary with uterine septum and review of the literatures | Human Reproduction | Ectopic Ovaries |
| Yalavarthi | 2017 | Mayer Rokitansky Kuster Hauser syndrome: Syndrome of Mullerian agenesis A report of two cases | Medical Journal of Dr. DY Patil University | Ectopic Ovaries |
| Yang | 2020 | PREPL Deficiency: A Homozygous Splice Site PREPL Mutation in a Patient With Congenital Myasthenic Syndrome and Absence of Ovaries and Hypoplasia of Uterus | Frontiers in Oncology | Ineligible Patient Population |
| Yang | 2014 | Irreducible inguinal hernia containing rudimentary uterine horn, ovary, and fallopian tube | Clinical and Experimental Obstetrics & Gynecology | Ectopic Ovaries |
| Yao | 2009 | Sonographic diagnosis of an ovary-containing inguinal hernia with the formation of a corpus luteum in an adult female | Ultrasound Obstet Gynecol | Ectopic Ovaries |
| Yildirim | 2006 | Ovarian agenesis and MURCS association. [Turkish] | Jinekoloji ve Obstetrik Dergisi | Non-english language |
| Yilmaz | 2019 | An approach to amenorrhea with three cases | Erciyes Medical Journal | Ineligible Patient Population |
| Yoshida | 2005 | Case of mature cystic teratoma of the greater omentum misdiagnosed as ovarian cyst | Journal of Obstetrics and Gynaecology Research | Ectopic Ovaries |
| Youn | 2012 | A case report: Ectopic ovary with a mature cystic teratoma diagnosed by laparoscopy | Journal of Minimally Invasive Gynecology | Ectopic Ovaries |
| Zarei | 2017 | Case study: Unilateral absence of the ovary and distal uterine tube without malformations of the uterus and/or urinary tract | Clinical Anatomy | Conference Abstract |
| Zarubina | 1978 | [Functional state of the pituitary gland-ovarian system in gonadal agenesis and dysgenesis] | Problemy Endokrinologii | Non-english language |
| Zhigang | 2007 | An intrarenal supernumerary ovary concurrent with a completely duplicated pelvis and ureter | International Urogynecology Journal | Ectopic Ovaries |
| Zhou | 2020 | Accessory ovary may be a treatment for infertility: Case report and review of current literatures | Journal of Obstetrics and Gynaecology Research | Ectopic Ovaries |
| Zhu | 2009 | Ultrasonographic diagnosis of ovary ectopic pregnancy followed birth control appliance being implanted | Chinese Journal of Medical Imaging Technology | Ectopic Ovaries |
| Zondek | 1989 | Unilateral renal agenesis in the female with special reference to associated malformations in the genital tract | Journal of Obstetrics and Gynaecology | Insufficient Information to Assess Ovarian Abnormality |
|  | 1952 | WEEKLY clinicopathological exercises; ectopic left kidney (in pelvis) with chronic pyelonephritis; congenital absence of right kidney, ovary & salpinx | The New England journal of medicine | Ectopic Ovaries |

**Supplementary Table 5: JBI Critical Appraisal Checklist for Case Reports**

| **First Authors Last Name** | **Q1. Were patient’s demographic characteristics clearly described?** | **Q2. Was the patient’s history clearly described and presented as a timeline?** | **Q3. Was the current clinical condition of the patient on presentation clearly described?** | **Q4. Were diagnostic tests or assessment methods and the results clearly described?** | **Q5. Was the intervention(s) or treatment procedure(s) clearly described?** | **Q6. Was the post-intervention clinical condition clearly described?** | **Q7.** **Were adverse events (harms) or unanticipated events identified and described?** | **Q8. Does the case report provide takeaway lessons?** | **Overall Appraisal** |
| --- | --- | --- | --- | --- | --- | --- | --- | --- | --- |
| Abargel et al. 2000^1^ | No | Yes | Yes | Yes | Yes | No | n/a | Yes | Include |
| Alexander 1947^2^ | Yes | Yes | Yes | Yes | Yes | No | n/a | No | Include |
| Ali et al. 1979^3^ | Yes | Yes | Yes | Yes | Yes | Yes | n/a | No | Include |
| Alrabeeah et al. 1988^4^ | Yes | Yes | Yes | Yes | Yes | Yes | n/a | Yes | Include |
| Alvir et al. 2013^5^ | Yes | Yes | Yes | Yes | Yes | No | n/a | Yes | Include |
| Aslam et al. 1995^6^ | No | Yes | Yes | Yes | Yes | Yes | n/a | Yes | Include |
| Awad et al. 2018^7^ | No | No | Yes | Yes | Yes | No | n/a | Yes | Include |
| Barsky et al. 2015^8^ | No | Yes | Yes | Yes | Yes | Yes | Yes | Yes | Include |
| Bates et al. 1982^9^ | Yes | Yes | Yes | Yes | Yes | Yes | Yes | Yes | Include |
| Bay et al. 2014^10^ | No | Yes | Yes | Yes | Yes | Yes | n/a | Yes | Include |
| Ben-Nun et al. 1988^11^ | No | Yes | Yes | Yes | Yes | Yes | Yes | Yes | Include |
| Bilij-Erski et al. 2019^12^ | No | Yes | Yes | Yes | Yes | Yes | n/a | Yes | Include |
| Blumberg et al. 1996^13^ | No | Yes | Yes | Yes | Yes | Yes | n/a | Yes | Include |
| Bousfiha et al. 2010^14^ | Yes | Yes | Yes | Yes | Yes | Yes | Yes | Yes | Include |
| Bradley et al. 1980^15^ | Yes | Yes | Yes | Yes | Yes | Yes | n/a | Yes | Include |
| Bugmann et al. 2001^16^ | No | Yes | Yes | Yes | Yes | Yes | n/a | Yes | Include |
| Burge 1958^17^ | No | Yes | Yes | Yes | Yes | Yes | n/a | Yes | Include |
| Castellani et al. 2013^18^ | No | Yes | Yes | Yes | Yes | Yes | n/a | Yes | Include |
| Chan et al. 1987^19^ | Yes | Yes | Yes | Yes | Yes | Yes | n/a | Yes | Include |
| Chen et al. 2014^20^ | No | Yes | Yes | Yes | Yes | No | n/a | Yes | Include |
| Choundhary et al. 2017^21^ | No | Yes | Yes | Yes | Yes | Yes | n/a | Yes | Include |
| Cucinella et al. 2013^22^ | No | Yes | Yes | Yes | Yes | No | n/a | Yes | Include |
| Currarino et al. 1989^23^ | No | Yes | Yes | Yes | Yes | No | n/a | Yes | Include |
| Dannreuther 1923^24^ | No | Yes | Yes | Yes | Yes | Yes | n/a | No | Include |
| Dare et al. 1989^25^ | No | Yes | Yes | Yes | No | No | n/a | Yes | Include |
| De et al. 2019^26^ | Yes | Yes | Yes | Yes | Yes | Yes | n/a | Yes | Include |
| Dede et al. 2008^27^ | Yes | Yes | Yes | Yes | Yes | Yes | n/a | Yes | Include |
| Demir et al. 2007^28^ | No | Yes | Yes | Yes | Yes | Yes | n/a | Yes | Include |
| Dueck et al. 2001^29^ | No | Yes | Yes | Yes | Yes | Yes | n/a | Yes | Include |
| Durous et al. 2019^30^ | No | No | Yes | No | No | Yes | n/a | Yes | Include |
| Eda et al. 2012^31^ | Yes | Yes | Yes | Yes | Yes | No | n/a | Yes | Include |
| Eustace 1992^32^ | No | No | Yes | Yes | Yes | Yes | n/a | Yes | Include |
| Fletcher et al. 1988^33^ | No | Yes | Yes | Yes | Yes | Yes | n/a | Yes | Include |
| Forceseneanu et al. 2013^34^ | No | Yes | Yes | Yes | Yes | Yes | n/a | Yes | Include |
| Galli et al. 2017^35^ | No | Yes | Yes | Yes | Yes | Yes | n/a | Yes | Include |
| Guthrie et al. 1909^36^ | No | Yes | Yes | Yes | Yes | No | n/a | Yes | Include |
| Gutierrez 1933^37^ | No | Yes | Yes | Yes | Yes | Yes | n/a | Yes | Include |
| Haydardedeoglu et al. 2006^38^ | No | Yes | Yes | Yes | Yes | No | n/a | Yes | Include |
| Kennedy et al. 1981^39^ | Yes | Yes | Yes | Yes | Yes | Yes | n/a | Yes | Include |
| Kent 1956^40^ | No | Yes | Yes | Yes | Yes | No | n/a | Yes | Include |
| Koh 1974^41^ | No | Yes | Yes | Yes | Yes | No | n/a | Yes | Include |
| Kriplani et al. 1995^42^ | No | Yes | Yes | Yes | Yes | Yes | n/a | Yes | Include |
| Kumar et al. 2007^43^ | Yes | Yes | Yes | Yes | Yes | Yes | n/a | Yes | Include |
| Kurcz et al. 1948^44^ | No | Yes | Yes | Yes | Yes | No | n/a | Yes | Include |
| Kusaka et al. 2007^45^ | Yes | Yes | Yes | Yes | Yes | No | n/a | No | Include |
| Lashgari 1975^46^ | Yes | Yes | Yes | Yes | Yes | Yes | n/a | Yes | Include |
| Lee et al. 2016^47^ | No | Yes | Yes | Yes | Yes | Yes | n/a | Yes | Include |
| Liu et al. 2013^48^ | No | Yes | Yes | Yes | Yes | No | n/a | Yes | Include |
| Loo et al. 2020^49^ | No | Yes | Yes | Yes | Yes | No | n/a | Yes | Include |
| Luvero et al. 2016^50^ | No | Yes | Yes | Yes | Yes | No | n/a | Yes | Include |
| Mamah et al. 2022 | No | Yes | Yes | Yes | Yes | No | n/a | Yes | Include |
| Mecklenburg et al. 1974^51^ | No | Yes | Yes | Yes | Yes | Yes | n/a | Yes | Include |
| Metoki et al. 1986^52^ | No | Yes | Yes | Yes | Yes | Yes | n/a | No | Include |
| Mishell 1938^53^ | No | Yes | Yes | Yes | Yes | Yes | n/a | Yes | Include |
| Morao et al. 2017^54^ | No | Yes | Yes | Yes | Yes | No | n/a | Yes | Include |
| Muppala et al. 2008^55^ | No | Yes | Yes | Yes | Yes | Yes | n/a | Yes | Include |
| Mutchinick et al. 2005^56^ | No | Yes | Yes | Yes | Yes | No | n/a | Yes | Include |
| Mylonas et al. 2003^57^ | No | Yes | Yes | Yes | Yes | Yes | n/a | Yes | Include |
| Okafor et al. 2013^58^ | Yes | Yes | Yes | Yes | Yes | Yes | n/a | Yes | Include |
| Ormonde et al. 2019^59^ | No | Yes | Yes | Yes | Yes | Yes | n/a | Yes | Include |
| Osmanagaoglu et al. 2010^60^ | No | No | Yes | Yes | Yes | No | n/a | Yes | Include |
| Pabuccu et al. 2011^61^ | No | Yes | Yes | Yes | Yes | No | n/a | Yes | Include |
| Peer et al. 1981^62^ | No | No | Yes | Yes | Yes | No | n/a | Yes | Include |
| Plevraki et al. 2004^63^ | No | Yes | Yes | Yes | Yes | Yes | n/a | Yes | Include |
| Rapisarda et al. 2009^64^ | No | Yes | Yes | Yes | Yes | No | n/a | Yes | Include |
| Rastogi et al. 2016^65^ | Yes | Yes | Yes | Yes | Yes | Yes | n/a | Yes | Include |
| Ruderman et al. 1962^66^ | Yes | Yes | Yes | Yes | Yes | Yes | Yes | Yes | Include |
| Siddigui et al. 2016^67^ | No | Yes | Yes | Yes | Yes | No | n/a | Yes | Include |
| Silva et al. 1995^68^ | Yes | Yes | Yes | Yes | Yes | No | n/a | Yes | Include |
| Simpson 1990^69^ | No | Yes | Yes | Yes | Yes | Yes | n/a | Yes | Include |
| Sinha 1983^70^ | No | Yes | Yes | Yes | Yes | Yes | n/a | Yes | Include |
| Sirisena 1978^71^ | Yes | Yes | Yes | Yes | Yes | Yes | n/a | Yes | Include |
| Sivanesaratnam 1986^72^ | Yes | Yes | Yes | Yes | Yes | Yes | n/a | Yes | Include |
| Stanojevic et al. 2000^73^ | Yes | Yes | Yes | Yes | Yes | Yes | Yes | Yes | Include |
| Suh et al. 2008^74^ | Yes | Yes | Yes | Yes | No | No | n/a | Yes | Include |
| Sukhadiya et al. 2014^75^ | Yes | Yes | Yes | Yes | Yes | Yes | n/a | Yes | Include |
| Sunku et al. 2015^76^ | No | Yes | Yes | Yes | Yes | Yes | n/a | Yes | Include |
| Surana et al. 1978^77^ | Yes | Yes | Yes | Yes | Yes | Yes | Yes | Yes | Include |
| Tanaka et al. 2013^78^ | No | Yes | Yes | Yes | Yes | Yes | n/a | Yes | Include |
| Tarry et al. 1986^79^ | No | Yes | Yes | Yes | No | No | n/a | Yes | Include |
| Taskin et al. 2013^80^ | Yes | Yes | Yes | Yes | Yes | No | n/a | Yes | Include |
| Topcu et al. 2013^81^ | No | Yes | Yes | Yes | Yes | Yes | n/a | Yes | Include |
| Turner et al. 2012^82^ | Yes | Yes | Yes | Yes | Yes | Yes | n/a | Yes | Include |
| Tzitzimikas et al. 2013^83^ | No | Yes | Yes | Yes | Yes | No | n/a | Yes | Include |
| Vaiarelli et al. 2012^84^ | No | Yes | Yes | Yes | Yes | Yes | Yes | Yes | Include |
| Varino et al. 1941^85^ | Yes | Yes | Yes | Yes | Yes | Yes | n/a | Yes | Include |
| Yazawa et al. 2019^86^ | No | Yes | Yes | Yes | Yes | Yes | n/a | Yes | Include |
| Yerebasmaz et al. 2016^87^ | No | Yes | Yes | Yes | Yes | No | n/a | Yes | Include |
| Zaitoon et al. 1982^88^ | Yes | Yes | Yes | Yes | Yes | Yes | n/a | Yes | Include |
| Zampieri et al. 2009^89^ | No | Yes | Yes | Yes | Yes | No | n/a | Yes | Include |
